# Chemical profile of *Juniperus excelsa* M. Bieb. essential oil within and between populations and its weed seed suppression effect

**DOI:** 10.1371/journal.pone.0294126

**Published:** 2024-02-08

**Authors:** Ivanka Semerdjieva, Valtcho D. Zheljazkov, Ivayla Dincheva, Tzenka Radoukova, Tess Astatkie, Vasilina Maneva, Dina Atanasova, Hafize Fidan, Stanko Stankov, Albena Stoyanova

**Affiliations:** 1 Department of Botany and Agrometeorology, Agricultural University, Mendeleev, Plovdiv, Bulgaria; 2 Department of Plant and Fungal Diversity and Resources, Institute of Biodiversity and Ecosystem Research, Bulgarian Academy of Sciences, Sofia, Bulgaria; 3 Department of Crop and Soil Science, Oregon State University, Corvallis, Oregon, United States of America; 4 Plant Genetic Research Group, AgroBioInstitute, Agricultural Academy, Sofia, Bulgaria; 5 Department of Botany and Biological Education, University of Plovdiv Paisii Hilendarski, Plovdiv, Bulgaria; 6 Faculty of Agriculture, Dalhousie University, Truro, Canada; 7 Plant Protection and Technology Department, Institute of Agriculture, Karnobat, Agricultural Academy, Karnobat, Bulgaria; 8 Department of Tourism and Culinary Management, Faculty of Economics, University of Food Technologies, Plovdiv, Bulgaria; 9 Department of Technology of Tobacco, Sugar, Vegetable and Essential Oils, University of Food Technologies, Maritza, Plovdiv, Bulgaria; Museu Paraense Emilio Goeldi, BRAZIL

## Abstract

The aims of this study were to (1) establish the intrapopulation and seasonal variation of *Juniperus excelsа* essential oil (EO); (2) compare the *J*. *excelsa* concrete and resinoid composition with its EO composition; and (3) investigate the potential herbicidal activity of *J*. *excelsa* EO against seeds of *Papaver rhoeas* L., *Consolida orientalis* (J.Gay) Schrödinger, *Anthemis arvensis* L., *Avena fatua* L., and *Agrostemma githago* L. Four independent studies were performed to meet these objectives. Twenty-eight individual trees were analyzed from two populations to establish intrapopulation and interpopulation variability of EOs yield and composition. In the seasonal dynamic study of leaf EO, samples from the same three trees and in the same population were collected in January, March, May, July, October, and December and their EO yield and composition determined. The EOs (intrapopulation and seasonal) were extracted by hydrodistillation, while the EO for the herbicidal test was obtained by steam distillation in a semi-commercial (SCom) apparatus. Overall, the EO yield varied significantly from 0.93% to 2.57%. *α*-Pinene (8.85–35.94%), limonene (11.81–50.08%), and cedrol (3.41–34.29%) were the predominant EO compounds in all samples (intrapopulation variability); however, *trans*-2,4-decadienol and *β*-caryophyllene were predominant in some individual trees. Four chemical groups were identified in the samples collected from two natural populations (intrapopulation). This is the first report on the compositions of *J*. *excelsa* concrete and resinoid. Cedrol (15.39%), 7-hydroxy-4-methyl-coumarin (17.63%), 1-octacosanol (36.85%), tritriacontane (16.08%), and tiacontanoic acid were the main compounds in the concrete and resinoid. *Juniperus excelsa* EO suppressed seed germination and seedling growth of *P*. *rhoeas*, *C*. *orientalis*, *A*. *arvensis*, *A*. *fatua*, and *A*. *githago*, demonstrating its potential to be used for the development of new biopesticides. The highest EO yield with high content of limonene and cedrol was obtained from samples harvested during the winter months (December, January, and March).

## 1. Introduction

*Juniperus excelsa* M. Bieb. (Greek juniper, Cupressaceae) is a conifer, evergreen species distributed throughout south-eastern Europe and south-western Asia [1]. In Bulgaria, the species is distributed across two floristic regions, namely the Struma river Valley and the Rhodope Mountains [2]. The habitats of *J*. *excelsа* are sclerophyllous type and extend along the steep slopes of deep, rocky, nutrient-poor gorges under a Mediterranean climate [2]. The distribution of *J*. *excelsa* in these dry, unfavorable conditions is very important for maintaining local ecosystems and reducing soil erosion [3]. Due to this environmental function and unique medieval forests (>100 years old) the populations of *J*. *excelsa* have a conservation significance. The Bulgarian habitats of *J*. *excelsa* were identified as the priority habitats in the European flora “39G3 Forests of Grecian juniper” included in Natura 2000 and they are the northernmost border distribution of this species [2, 4, 5]. There are genetic studies on woody plant populations distributed in the eastern Mediterranean climate, including on *J*. *excelsa* [6, 7]. These studies showed high levels of genetic diversity at population level and within a population of *J*. *excelsa* [6, 7]. High variability was found in morphological characteristics (leaves, cones, woods) of *J*. *excelsa* distributed in Bulgarian populations [1]. The latter authors found that Bulgarian samples of *J*. *excelsa* had different morphological characteristics than Crimean samples [1]. Furthermore, the authors assumed that the Bulgarian population originated from another Pleistocene refuge and differed from species distributed in Crimea [1]. All *J*. *excelsa* trees contain an aromatic EO; however, given the genetic diversity within the species, there is a need to assess the variability of EO composition of individual trees within a population. Furthermore, it is not clear how the quantitative and qualitative composition of the EOs change during different seasons. Until now, there has been no study on intrapopulation EOs variability of individual trees. Most of the published records did not clarify whether the samples were pooled from several trees or from a single tree (Table 1). Therefore, published research findings to date did not clarify the variability in EO composition of individual trees within the species. Previous reports on *J*. *excelsa* EOs from other countries and Bulgaria have shown high dissimilarities as summarized in Table 1. Most studies of *J*. *excelsa* EO were from Iran, Lebanon, and Pakistan (Table 1), while relatively few were from Greece, Bulgaria, Turkey, and the Republic of Macedonia [5, 8, 9]. In previous reports, only one or two samples per population of *J*. *excelsа* were studied, which may have misrepresented the existing diversity in EO profile within and between populations. Usually, the factors that influence phytochemical variation of EOs are thought to be the plant ontogeny, genetic variations, environmental conditions, and harvest season [10, 11].

**Table 1:** Literature data of essential oils composition of *Juniperus excelsa*.

Origin	Analyzed part	DT	Oil yield	The main compounds (%)	Reference
**Bulgaria**	twigs	HD/SD	0.69–1.87%	*α*-pinene (18.90–22.30); *α*-limonene (23.70–27.50); *α*-cedrol (24.06–27.0);	[5]
**R. Macedonia**	leaves, berries	SD	1.6–9.4 ml/kg (berries); 8.9–13.9 ml/kg (leaves)	Dojran—*α*-pinene (70.81, berries, 33.83, leaves); limonene (4.0, berries; 6.14, leaves); Ohrid—*α*-pinene (4.51–7.09, berries; 1.76–2.59, leaves); sabinene (58.85–62.58, berries; 28.52–29.49, leaves); *β*- myrcene (5.06–5.65, berries; 2.09–2.78, leaves);	[8]
**Turkey**	berries		no	*α*-pinene (46.1);	[12]
**Iran**	leaves	HD	0.5%	*α*-pinene (67.71); *α*-cedral (11.5); *δ*-3-carene (5.19); limonene (4.41);	[14]
**Iran**	leaves	HD	0.08%–3.78% (v/w)	*α*-pinene (12.05–90.09); limonene (0.6–9.1%); *β*-myrcene (0.6–5.5%); *α*-cedrol (0.0–29.5%).	[15]
**Iran**	leaves; berries	SD	no	*α*-pinene (32.72–59.90, leaves; 78.26, berries); *ß*-pinene (2.63–15.83); 1,4-cineole (6.50–6.79, leaves; 6.97, leaves); limonene (7.02–9.73, leaves; 0.21, berries); (*E*)-*ß*-ocimene (1.82–5.54, leaves; 3.48, berries); bergamal (0.11–5.05, leaves).	[18]
**Greek**	leaves	HD		cedrol (28.1); *α*-pinene (22.5); limonene (22.7)	[27]
**R. Macedonia**	berries	HD	1.2%	sabinene (72.8)	[28]
**Turkey**	leaves, berries	HD	no	*α*-pinene (34.0, berries; 29.7, leaves); cedrol (12.3, berries; 25.3, leaves)	[29]
**Iran**	leaves, berries	petroleum ether	1.55–1.60%	*α*-pinene (47.64–32.34); α-cedrol (12.01–3.06%); myrcene (5.91–5.40).	[30]
**Iran**	leaves, berries	SD	0.5%;0.6%;0.85%	*α*-pinene (67.3;14.2;56.6), leaves; *α*-pinene (15.2;75.6;57.2), berries	[31]
**Pakistan**	berries	solvent	5.8, 6.5, 4.5%, n-tetradecane	*α*-pinene (1.70–15.92); phyllocladene,(-)-(2.57–10.73); cedrol (0.0–8.63); pimara-8(14),15-diene (3.92–5.85);	[32]
**Oman**	berries	HD	0.27%	*α*-terpinene (23.85); limonene (23.42); fenchene (6.57); camphene (6.0); *β*-3-carene (4.17); 4-terpineol (2.93); germacrene B (2.21); myrcene (1.96); *α*-pinene (1.77); *β*-pinene (1.53); abietatriene (1.13);	[33]
**Lebanon**	berries	HD	1.17%	*α*-pinene (86.8); myrcene (3.2);	[34]
**Lebanon**	leaves, berries, twigs	HD	no	*α*-pinene (6.9–68.9, leaves; 78.3–89.8, twigs; 75–95.2 berries); *δ*-3-carene (3.3–22.1, leaves; 1.1–2.6, twigs; 0.9–2.4, berries); *α*-cedrol (8.7–57.0, leaves; 1.2–8.1, twigs, 0.0–4.8, berries);	[35]
**Bulgaria**	berries	HD	1.22%	*α*-pinene (52.4); *β*-pinene (3.08); *β*-myrcene (3.67); limonene (7.07); germacrene D (4.2);	[36]
**Iran**	leaves	HD	4.3% v/w	*α*-pinene (73.27); *α*-cedrol (5.53).	[37]

HD–hydrodistillation; SD–steam distillation; DT–distillation type.

A review of published research revealed that different plant parts of *J*. *excelsа* were found to contain relatively high amounts of EO (Table 1). The EO composition of *J*. *excelsa* leaves and berries (female cones) has been intensively studied in the last decade [5, 8, 9, 12–18]. Furthermore, extracts and EOs of *J*. *excelsa* have been documented to exhibit promising biological effects as antioxidant, anti-inflammatory, antimicrobial, insecticidal, and herbicidal activities [5, 8, 9, 12–14, 16, 19, 20]. Certified organic production system prohibits the use of chemical pesticides; therefore, natural products have been widely studied for weed control in such systems. The EOs are known for their phytotoxicity and some EOs were shown to inhibit weed seed germination by altering the enzymes’ activities, membrane permeability, and respiration [21, 22]. *Juniperus* species are known for their high EO content, mainly monoterpenes, which may have allelopathic effects [22]. Generally, the benefits of EOs as allelochemicals have been extensively studied during the past decades [19, 23–26], but such studies on *J*. *excelsa* EO is scarce.

Until now, there were no intrapopulation and comprehensive seasonal studies evaluating *J*. *excelsа* EOs. Furthermore, the chemical compositions of *J*. *excelsa* concrete and resinoids have not been reported. Consequently, this research gap guided the objectives of this study to be: (1) to assess the intrapopulation and seasonal variation of *J*. *excelsа* EO content and composition; (2) to compare concrete and resinoid with the EO composition of *J*. *excelsa*; and (3) to investigate the potential herbicidal effects of EO against the seed of five weed species. The working hypothesis of this study was that the EO content and composition would be similar among different trees in populations and during different seasons. Furthermore, the *J*. *excelsa* essential oil would have bioherbicidal effect on the germination of weed species.

## 2. Material and methods

### 2.1. Collection of the plant material

#### 2.1.1. Intrapopulation variation of Juniperus excelsa EO content and composition

Samples of *Juniperus excelsa* were collected in January, 2020 from two natural populations (S1 Fig); the first population is the reserve “Tisata” that is spread in two mountains, the East Pirin Mountain (in Tables is as location 1) and in the Maleshevska Mountain (in Tables is as location 2), and the second population is the reserve Izgoryaloto Gyune, Krichim in the Rhodope Mountains (in Tables as location 3). According to the geographical location, the reserve “Tisata” includes the eastern parts of Pirin Mountain and the parts of Maleshevska Mountain, and we have collected samples from both parts of the reserve–East Pirin Mountain and Maleshevska Mountain. The numbers of collected samples for individual trees were as follows: (1) samples from 6 trees in East Pirin Mountain (part of the reserve “Tisata”) (location 1); (2) samples from 12 trees in Maleshevska Mountain (part of the reserve “Tisata”) (location 2); and (3) samples from 10 trees in the reserve “Izgoryaloto Gyune” (location 3). A total of 28 samples from individual trees were analyzed, with two replicates for each sample. The coordinates and the altitudes are presented in S1 Table.

Prior to sample collection, we received a special permit from the directorate of the Pirin park and the Bulgarian Ministry of the Environment (736/12 March 2018, issued to Dr. Tzenka Radoukova and Dr. Valtcho D. Zheljazkov). The small branches of *J*. *excelsa* samples were deposited at the herbarium at Agricultural University, Plovdiv, Bulgaria, SOA [38].

#### 2.1.2. The seasonal variation in J. excelsa EO content and composition

Samples for the seasonal variation study were collected from the reserve Izgoryaloto Gyune, Krichim. Three trees were marked, and all the samples were collected from the same trees in January, March, May, July, October, and December 2020. A total of six samples per tree per collection event were obtained and therefore, there were 18 samples extracted and analyzed in this seasonal variation study.

### 2.2. Methods

#### 2.2.1. Essential Oil (EO) extraction of *Juniperus excelsa*

*2*.*2*.*1*.*1*. *Intrapopulation variability of essential oil (EO)*. All *J*. *excelsa* samples that were collected from the two natural populations (3 locations); (1) the reserve “Tisata” (2 locations), and (2) the reserve “Izgoryaloto Gyune”, Krichim were extracted by hydrodistillation for 3 h using Clevenger-type apparatus modified by Balinova and Diakov [39]. The modification is in the length of the glass tube from the flask containing the raw material with water to the condenser. It is a reflux. With this modification, the path is almost 1.5 times shorter. In addition, the glass tube through which the distillation waters return to the flask and re-distill is closer to the flask. Overall, a total of 28 samples from individual trees were analyzed, with two repetitions each. Each sample was 100 g leaves without berries. Immediately prior to the distillation, the subsamples were cut into 5 mm pieces. We used 1000 mL water for each sample so the ratio of the biomass to water was 1:10. At the end of the distillation, the EO was dried over anhydrous sulfate and stored in tightly closed dark vials at 4°C until they could be analyzed for their chemical profile. The EO yields are reported on an absolute dry weight.

*2*.*2*.*1*.*2*. *Seasonal variability of essential oil (EO) content and composition*. The *J*. *excelsa* samples used for the seasonal variability of EO study were dry in laboratory in the Department of Botany and Agrometeorology at the Agricultural University, Plovdiv. All samples were ground in water immediately prior to the distillation and they were extracted by hydrodistillation for 2 h using Clevenger-type apparatus following the procedure outlined in the Russian Pharmacopoeia [40], in two replicates. Each sample consisted of 100 g leaves without berries plus 1200 mL water resulting in a ratio of 1:12. The EO was separated from water at the end of each distillation, measured on an analytical scale, and kept in a freezer until the gas chromatography (GC) analyses could be performed. In this study for seasonal variability of *J*. *excelsa* EO we report the EO as gram [g] per air-dried biomass weight.

#### 2.2.2. Obtaining resenoids and concrete

*2*.*2*.*2*.*1*. *Juniperus excelsa concrete*. *Juniperus excelsa* concrete was obtained by static extraction with the following technological parameters: the 50–70 g samples of leaves were cut into small pieces and placed in a 50 mL Becher cup. Then *n*-hexane (Sigma-Aldrich, Steinheim, Germany) was added in a ratio of 1:6 as extractant. Becher cups were covered with aluminum foil and placed in a drying cabinet at 40°C temperature following the method of Stoyanova et al. [41]. Twofold batch extraction was made for 3 h and 1 h each. The solvent was completely removed by evaporation on a rotary vacuum evaporator IKA*HB eco at a water bath temperature of 35°C according to the method described by Stoyanova et al. [41].

*2*.*2*.*2*.*2*. *Juniperus excelsa resinoid*. *Juniperus excelsa* resinoid was obtained by static extraction with the following technological parameters: samples of 50–70 g of leaves were cut into small pieces and placed in a 50 mL Becher cup. After that, 95% ethanol (FILLAB, Bulgaria)) was added in a ratio of 1:6 as extractant. Becher cups were covered with aluminum foil and placed in a drying cabinet at 60°C temperature following the method of Stoyanova et al. [41]. Twofold batch extraction was used for 4 h and 2 h. The solvent was completely removed by evaporation on a rotary vacuum evaporator IKA*HB eco at a water bath temperature of 55°C according to Stoyanova et al. [41].

#### 2.2.3. Gas Chromatography-Mass Spectrometry and Flame Ionization Detection (GC-MS-FID) of essential oils and the concrete and resinoid

A GC-MS analysis of EOs, concrete and resinoid was carried out on a 7890A gas chromatograph (Agilent Technologies) interfaced with a 5975 C mass selective detector (Agilent Technologies). Separations were performed using a 30 m × 0.25 mm (i.d.) DB-5ms column coated with (5%-phenyl)-(95%)-methylpolysiloxane as a stationary phase. The flow rate of carrier gas (Helium) was maintained at 1.0 mL/min. The injector and the transfer line temperature were kept at 250°C. The oven temperature program used was 60°C for 5 min then 5°C/min to 300°C for 10 min. The injection volume was 1.0 μL in a split mode ratio 10:1. The mass spectrometer was scanned from 50 to 550 m/z. All mass spectra were acquired in electron ionization (EI) mode with 70 eV. In order to calculate the retention index RI of each compound, a mixture of aliphatic hydrocarbons (C_8_-C_40_, Sigma) was injected into the system under the above temperature program. The identification of the components was obtained by comparing the RI and the spectral data from NIST’08 [42, 43]. Before analysis, samples of 10.0 mg of concrete and resinoid were dissolved in 1.0 mL *n*-hexane and 1.0 mL absolute ethanol, respectively. Both solutions were filtered before chromatographic separation.

#### 2.2.4. The test for herbicidal effects of EO against seeds of Papaver rhoeas, Consolida orientalis, Anthemis arvensis, Avena fatua, Agrostemma githago and plant material and essential oil (EO) extraction and analyses

Seeds of *P*. *rhoeas*, *C*. *orientalis*, *A*. *arvensis*, *A*. *fatua*, *A*. *githago* weed species were studied in this experiment. The weed seeds were collected from mature plants from the experimental fields of the Institute of Agriculture, Karnobat in 2021 according to the Guidelines for laboratory studies on germination ecology [44]. Only healthy-looking weed seeds were selected. The collected seeds were stored at 4°C until the herbicidal test was conducted.

*2*.*2*.*4*.*1*. *Tetrazolium test (TT) and water imbibition of Papaver rhoeas*, *Consolida orientalis*, *Anthemis arvensis*, *Avena fatua*, *Agrostemma githago seeds*. The tetrazolium test was used to assess seed viability of target weed species and it was conducted in laboratory in the Department of Botany and Agrometeorology at the Agricultural University, Plovdiv. To establish the permeability of the seed coat was conducted imbibition water of weed seeds, because the permeability of the seed coat is a very important characteristic of seed germination. Imbibition of water of target seeds was conducted according to methodology described by Baskin and Baskin [44]. To ascertain the absorption of water by seeds, the dry seeds of the target species were weighed and submerged in water. Their weight was measured after 60 and 120 minutes. If their weight increased, it implies that the seed coat is permeable and capable of absorbing water [44]. Since we had prior knowledge that *A*. *arvensis* germinated, we excluded it from the water imbibition experiment. As described in Materials and methods (2.2.4.), the seeds were collected from mature plants. For TT was used 50 seeds per species. Prior to the test the weed seeds were soaked in water in Petri dishes (100 × 15 mm) for 24 h at 30–35°C. Then, the seeds were cut. After that, 1% solution of 2,3,5-triphenyltetrazolium chloride was used according to Peters [45]. According to the requirements of the methods [45], the solution of 2,3,5-triphenyltetrazolium chloride was used in different amounts (depending on the size of the seeds) so as to cover the seeds and allow its absorption. All tests were conducted with three replicates. The interpretations of results were according to the official association of the Tetrazolium Subcommittee of the Association of Official Seed Analysts [45]. In the beginning the tetrazolium solution was colorless but after respiration process of the seeds its color changed to red. Viable seeds are those whose embryos are colored red.

*2*.*2*.*4*.*2*. *Essential oil (EO) extraction and analyses for herbicidal test*. Essential oil of *J*. *excelsa* for potential herbicidal test was conducted in semi-commercial (SCom) steam extractor as previously reported [5]. The quality of the composition of EO was also reported [5].

*2*.*2*.*4*.*3*. *The method for testing herbicidal effect*. The test was carried out at the laboratory of Institute of Agriculture in Karnobat in 2021. The EO of *J*. *excelsa* semi-commercial steam distillation was tested at 0 μL (control), 5 μL, 10 μL, and 20 μL concentrations, in three replicates. The assay of EO for potential herbicidal effect was evaluated in Petri dishes as a previously described [19, 24]. Twenty seeds from each of *P*. *rhoeas*, *C*. *orientalis*, *A*. *arvensis*, *A*. *fatua*, and *A*. *githago* were arranged in Petri dishes (100 × 15 mm) between three layers of filter paper wetted with 5 mL of distilled water. The EO was attached once to upper side of a filter paper of the Petri dishes. After that, Petri dishes were sealed with Parafilm and incubated in a thermostat (Binder GmbH, Tuttlingen, Germany) at a permanent temperature of 22°C as reported previously [19]. On the 4^th^ day, the germination energy (%) of *P*. *rhoeas*, *C*. *orientalis*, *A*. *arvensis*, *A*. *fatua*, and *A*. *githago* was measured, and on the 7^th^ day the germination (%), sprout length (cm), and root length (cm) of germinated seeds were measured according to the ISTA standard [46]. Descriptive statistics were calculated for the measured seed germination related variables (germination energy [%]; the germination [%], sprout length [cm], and root length [cm]).

### 2.3. Statistical analyses

#### 2.3.1. The intrapopulation variation of *J*. *excelsa* Eos

The effect of Location (East Pirin [location 1], Malesjevska mountain [location 2], and Krichim “Izgoryaloto Gune” [location 3]) and Tree (1–6 at East Pirin, 1–12 at Malesjevska Mountain, and 1–10 at Krichim “Izgoryaloto Gune”) nested in Location on 19 response variables (oil yield, *α*-pinene, limonene, *trans*-2,4-decadienol, *β*-caryophyllene, *β*-cedrene, *δ*-cadinene, caryophyllene oxide, *allo*-cedrol, cedrol, 1,10-di-epi-cubenol, cubenol, aliphatic hydrocarbons, monoterpene hydrocarbons, oxygenated monoterpenes, sesquiterpene hydrocarbons, oxygenated sesquiterpenes, aromatic hydrocarbons, and oxygenated aromatics) was determined using a Nested design with the two effects in the model being Location and Tree (Location). The analysis of variance (ANOVA) was done using Proc Mixed of SAS [47]. When the effect of Tree (Location) is significant, multiple means comparison (MMC) was done on the 28 trees from the three locations. Letter groupings were done by conducting MMC using the least squares means, which is equivalent to the LSD method at the 1% level of significance to reduce the over inflation of Type II experiment twise error rate.

For each response variable, normal distribution and constant variance assumptions on the error terms were validated as described in Montgomery [48], and an appropriate transformation was applied on some of the response variables where the assumption was violated; however, the results in the Tables are presented after back transforming them to the original scale. Since the effects of Location and Tree (Location) on oxygenated monoterpenes and aromatics hydrocarbons were not significant, multiple means comparison was not performed. Instead, the overall means were calculated to be 3.9% and 0.21%, respectively. To determine the similarity level of the locations in terms of all 11 constituents (*α*-pinene, limonene, *trans*-2,4-decadienol, *β*-caryophyllene, *β*-cedrene, *δ*-cadinene, caryophyllene oxide, *allo*-cedrol, cedrol, 1,10-di-epi-cubenol, and cubenol), cluster analysis (complete linkage clustering) was conducted to generate a dendrogram as described in Johnson and Wichern [49]. In this study, cluster analysis, which is a part of multivariate data analysis, is used because it allows the identification of the locations and the trees that share similar EO constituents. These similarities are represented graphically using a dendrogram.

#### 2.3.2. Seasonal dynamics of *J*. *excelsa* Eos

The effect of Tree (3 levels: Tree1, Tree2, and Tree3) on oil yield, *α*-pinene, *p*-cymene, limonene, *α*-cedrene, *β*-cedrene, cedrol, *allo*-cedrol, monoterpenes, and sesquiterpenes whose values were measured from the same tree repeatedly in January, March, May, July, October, and December was determined by doing Repeated Measures Analysis (RMA). RMA allows the determination of the effect of Tree, and how it evolves during these measurement months. Compound Symmetry (CS) covariance structure was identified as the most appropriate one by using Akaike Information Criterion [50]. The RMA was completed using the Mixed Procedure of SAS [47], and further multiple means comparison was completed for significant (*p*-value < 0.05) or marginally significant (0.05 < *p*-value < 0.1) effects by comparing the least squares means of the corresponding treatment combinations. Since the interaction effect of Tree and Month was significant on the 9 response variables, and marginally significant on one (Oil yield) response variable, letter groupings were generated using a 5% level of significance. For each response variable, the validity of model assumptions (normal distribution and constant variance assumptions on the error terms) was verified by examining the residuals as described in Montgomery [48].

## 3. Results

### 3.1. Essential oil yield

#### 3.1.1. Intrapopulation and interpopulation variability of yield

The results of variability of EO yield are presented in Tables 2 and 3, respectively. As we mentioned above, two natural populations were studied namely the reserve “Tisata” and the reserve “Izgoryaloto Gyune”, Krichim in the Rhodope Mountains. According to the statistical analysis, the highest EO yield (2.57%) was received from the reserve “Tisata”, Malesjevska mountain, tree 12 (Table 3). Considerable variation in the EO yield was found among two populations and between individual trees from 0.93% to 2.57%, respectively (Table 3). This variation of results of oil yield was statistically significant and it is presented in Tables 2 and 3. For example, in some of the samples collected from Eastern Pirin, “Tisata” (location 1), the EO yield was 0.93%, while in other trees of the same location, the EO yield was 2.04% (Table 3). A similar variability in EO content was found for samples collected in the western part of the reserve “Tisata” (Malesevska Mountain, location 2) (1.03%–2.57%) and the reserve “Izgoryaloto Gyune”, Krichim (2^nd^ population, location 3) (1.29%–2.34%) (Table 3).

**Table 2:** ANOVA *p*-values that show the significance of the effects of location and tree (Location) on 19 response variables of *Juniperus excelsa* in Bulgaria. Significant effects that need multiple means comparison are shown in bold.

SV	Oil yield	*α*-Pinene	Limonene	*trans*-2,4-Decadienol	*β*-Caryophyllene	*β*-Cedrene	*δ*-Cadinene
**L**	0.001	0.001	0.001	0.001	0.001	0.001	0.001
**T(L)**	**0.001**	**0.001**	**0.001**	**0.001**	**0.001**	**0.001**	**0.001**
**SV**	caryophyllene oxide	*allo*-cedrol	cedrol	1,10-di-epi-cubenol	cubenol	Al.Hy.	M.Hy.
**L**	0.001	0.001	0.001	0.001	0.001	**0.003**	**0.002**
**T(L)**	**0.001**	**0.001**	**0.001**	**0.001**	**0.001**	0.926	0.710
**SV**	Ox.M.	S.Hy.	Ox.S.	A. Hy.	Ox.A.		
**L**	0.275	**0.001**	**0.049**	0.383	0.725		
**T(L)**	0.908	0.937	0.684	**0.072**	0.519		

SV = source of variation, L = location, T = tree, Ox = oxygenated, Hy = hydrocarbons; M = monoterpenes; S = sesquiterpenes; A = aromatic; Al = aliphatic.

**Table 3:** Mean concentration (%) of oil yield, *α*-pinene, limonene, *trans*-2,4-decadienol, cedrol and *β*-caryophyllene of intrapopulation and interpopulation variability of essential oil of *Juniperus excelsa* in Bulgaria obtained from 28 trees collected at two population reserve “Tisata” (East Pirin, location 1 and Malesjevska mountain, location 2) and the reserve “Izgoryaloto Gyune”, Krichim (location 3). a-d = abcd. Within each column, means sharing the same letter are not significantly different.

Tree	Oil yield	*α*-Pinene	Limonene	*trans*-2,4 Decadienol	*β*-Caryophyllene	Cedrol
**Population 1, Location 1**						
Tree1(EPT)	2.04 a-d	33.96 ab	21.03 m	1.84 hi	2.14 i-m	17.12 gh
Tree2(EPT)	0.93 i	18.13 kl	22.12 klm	2.15 fgh	3.09 c-f	24.31ef
Tree3(EPT)	1.62 c-h	20.94 ij	32.15 e	1.63 ij	2.82d-k	19.22 g
Tree4(EPT)	1.22 f-i	27.07 d	13.37 p	2.56 ef	3.22 cd	29.25 b
Tree5(EPT)	1.44 e-i	16.08 mn	33.91 e	3.04 cd	2.97 c-h	16.18 h
Tree6(EPT)	1.29 f-i	13.90 o	31.49 ef	4.05 b	2.84 c-j	25.03 ef
**Population 1, Location 2**						
Tree1(MMT)	1.79 b-f	22.84 ghi	28.14 gh	2.05 ghi	3.46 c	23.23 f
Tree2(MMT)	1.53 d-h	16.79 lm	26.05 hi	0.56 lmn	2.99 c-h	33.00 a
Tree3(MMT)	1.35 e-i	15.23 mno	38.29 cd	0.29 n	2.48 e-m	24.30 ef
Tree4(MMT)	1.12 ghi	25.11 def	24.67 ij	1.01 kl	4.04 b	24.30 ef
Tree5(MMT)	1.71 c-g	13.85 o	23.71 jkl	0.44 mn	3.05 c-g	34.29 a
Tree6(MMT)	1.44 e-i	29.77 c	15.18 o	0.14 n	2.36 g-m	29.01 b
Tree7(MMT)	1.03 hi	26.68 de	16.84 n	0.32 n	4.76 a	25.98 de
Tree8(MMT)	1.09 hi	23.26 fgh	21.78 lm	2.49 efg	2.91 c-i	25.64 de
Tree9(MMT)	1.51 d-i	14.99 no	37.97 cd	1.64 ij	3.17 cde	24.32 ef
Tree10(MMT)	1.23 f-i	35.94 a	24.16 ijk	0.31 n	4.66 a	3.41 i
Tree11(MMT)	2.07 a-d	20.06 jk	27.03 gh	0.88 klm	2.57 d-m	29.05 b
Tree12(MMT)	2.57 a	8.85 r	40.31 c	1.75 hi	2.69 d-l	27.09cd
**Population 2, Location 3**						
Tree1(IG)	2.05 a-d	14.88 no	37.00 d	0.31 n	1.91 lm	25.03 ef
Tree2(IG)	2.16 abc	21.17 hij	24.22 ij	4.51 a	2.28 h-m	26.19 cde
Tree3(IG)	1.62 c-h	34.12 ab	11.81 q	0.53 mn	3.13 c-f	26.13 cde
Tree4(IG)	2.12 abc	10.73 q	50.08 a	1.69 i	1.87 lm	15.23 h
Tree5(IG)	1.73 c-f	16.37 lmn	39.96 cd	1.77 hi	2.13 i-m	18.03 gh
Tree6(IG)	1.29 f-i	32.30 bc	17.24 n	1.20 jk	1.78 m	23.43 f
Tree7(IG)	1.92 b-e	12.18 p	45.20 b	3.37 c	1.73 m	16.75 gh
Tree8(IG)	1.50 d-i	26.16 de	29.14 fg	0.32 n	2.45 f-m	17.07 gh
Tree9(IG)	2.34 ab	11.05 pq	34.01 e	2.83 de	2.06 j-m	28.00 bc
Tree10(IG)	1.60 c-h	24.45 efg	39.95 cd	1.20 jk	2.02 klm	16.04 h

EPT—East Pirin, Tisata; MMT—Malesjevska Mountain, Tisata; IG—Izgoryaloto Gyune.

#### 3.1.2. Seasonal variability of yield

**Fig 1:**
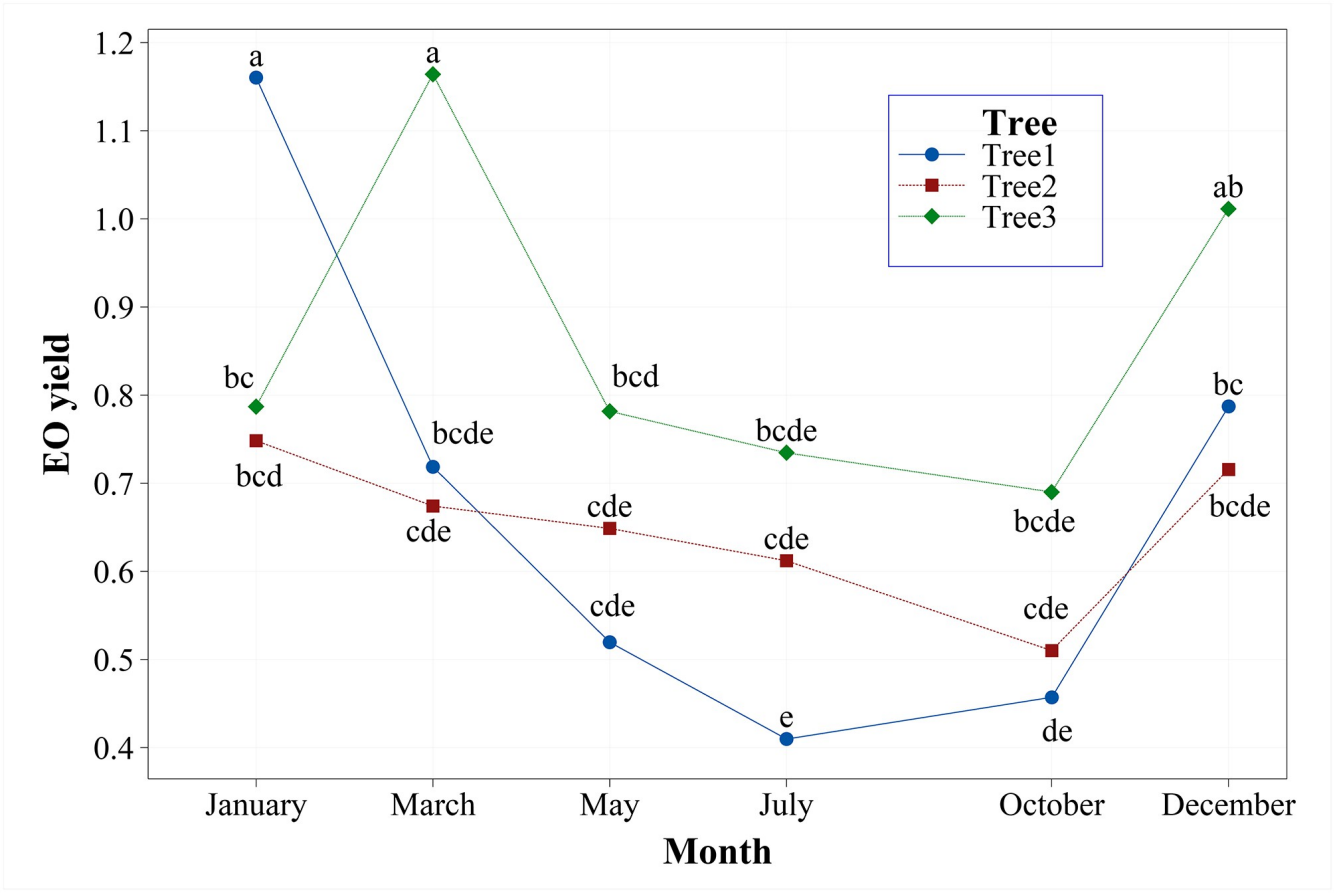
Interaction effect of month and tree on EO yield that shows the seasonal dynamics of EO yield of *Juniperus excelsa*.

The seasonal dynamics of EO yield are presented in Tables 4 and 5, and Fig 1. The results indicated that the EO yield depends primarily on the genetics and then on the harvesting season (Fig 1). For example, the EO yield of Tree 1 was significantly higher in samples collected in January (1.16%, Table 5), while the EO yield of Tree 3 was higher in samples collected in March (1.16%, Table 5; Fig 1). The samples of Tree 2 showed high EO yield in January and December (Table 5). Statistical analyses results showed significant main and interaction effects of individual trees and the month of collection (Table 4). Generally, the best period for harvesting *J*. *excelsa* samples with a high EO yield was January or March. Our data showed that the oil yield was low in May, July, and October (Table 5).

**Table 4:** ANOVA *p*-values that show the significance of the main and interaction effects of Tree and Month on 10 response variables of *Juniperus excelsa* in Bulgaria (seasonal variability). Significant effects that require multiple means comparison are shown in bold.

Source of variation	Oil yield	*α*-Pinene	*p*-Cymene	Limo nene	*α*-Cedrene	*ß*-Cedrene	Cedrol	*allo*-Cedrol	Monoterpenes	Sesquiterpenes
Tree	0.007	0.001	0.001	0.001	0.001	0.001	0.001	0.001	0.003	0.001
Month	0.003	0.013	0.019	0.001	0.001	0.001	0.001	0.001	0.001	0.001
Tree*Month	**0.075**	**0.001**	**0.031**	**0.001**	**0.001**	**0.001**	**0.001**	**0.001**	**0.001**	**0.001**

**Table 5:** Mean concentration (%) of seasonal variability of oil yield, *α*-pinene, *p*-cymene, limonene, *α*-cedrene, *β*-cedrene, cedrol, allo-cedrol, monoterpenes, and sesquiterpenes of *Juniperus excelsa* in Bulgaria obtained from 3 trees collected at 6 different months. Within each column, means sharing the same letter are not significantly different.

Month	Oil yield	*α*-Pinene	*p*-Cymene	Limonene	*α*-Cedrene	*β*-Cedrene	Cedrol	*allo*-Cedrol	MT	ST
**Tree1**										
Jan	1.16 a	21.2 de	1.45 cde	26.97 cde	2.87 bcd	1.60 de	31.68 a-d	2.1ef g	66.58 b	3.04 d
March	0.72 b-e	15.76 h	1.92 a	29.11 bc	3.21 ab	1.01 hi	35.42 a	3.06 bc	54.02 j	3.14 a
May	0.52 cde	17.86 fgh	1.95 a	28.91 bc	1.86 gh	0.85 i	27.39 ef	3.01 bc	61.76 c-f	2.96 ef
July	0.41 e	18.44 fg	1.61 bcd	26.67 cde	1.81 gh	1.64 de	30.86 bcd	3.34 ab	58.67 gh	3.04 d
Oct	0.46 de	18.15 fgh	1.71 abc	23.33 gh	1.90 gh	1.14 gh	34.29 abc	3.54 a	55.89 hij	3.10 abc
Dec	0.79 bc	18.17 fg	1.78 ab	33.13 a	2.05 fg	0.92 i	27.34 ef	2.23 ef	63.37 cd	2.93 fg
**Tree2**										
Jan	0.75 bcd	22.37 cde	0.97 f	24.25 fgh	1.96 gh	1.71 cd	31.78 a-d	1.96 fgh	59.22 fg	3.03 d
March	0.67 cde	25.79 ab	0.91 f	26.27 def	2.49 de	1.55 def	29.81 de	1.53 i	60.66 d-g	2.96 ef
May	0.65 cde	24.73 abc	0.97 f	22.30 hi	2.36 ef	1.47 ef	30.66 cde	1.93 fgh	58.58 ghi	3.04 d
July	0.61 cde	26.68 a	1.02 f	25.04 efg	2.88 bcd	2.01 ab	25.09 f	2.74 cd	63.27 cde	2.93 fg
Oct	0.51 cde	23.87 bc	0.88 f	20.33 i	2.59 cde	2.08 a	34.58 ab	1.74 ghi	54.31 j	3.13 a
Dec	0.72 bcde	23.25 cd	0.99 f	25.66 d-g	2.72 cde	1.87 bc	29.67 de	1.69 hi	58.22 ghi	3.04 d
**Tree3**										
Jan	0.79 bc	16.15 gh	1.55 bcde	31.40 ab	1.61 h	0.85 i	33.75 abc	2.39 de	58.32 ghi	3.05 cd
March	1.16 a	18.17 fg	1.35 de	25.10 efg	2.98 bc	1.34 fg	34.47 ab	2.62 d	55.75 ij	3.11 ab
May	0.78 bcd	22.71 cd	1.77 ab	27.86 cd	2.97 bc	1.43 ef	25.34 f	2.62 d	64.49 bc	2.99 g
July	0.73 b-e	20.11 ef	1.68 abc	26.06 def	3.23 ab	1.48 ef	31.81 a-d	2.70 cd	57.99 ghi	3.05 bcd
Oct	0.69 b-e	22.46 cde	1.33 e	27.91 cd	2.85 bcd	1.60 de	29.70 de	2.17 ef	60.41 efg	2.99 de
Dec	1.01 ab	24.11 bc	1.79 ab	31.49 ab	3.40 a	1.52 def	18.71 g	1.55 i	71.19 a	2.72 h

MT–monoterpenes; ST–sesquiterpenes; b-e = bcde; a-d = abcd; c-f = cdef; d-g = defg

### 3.2. Essential Oil (EO) compositions of *Juniperus excelsa*

#### 3.2.1. Intrapopulation and interpopulation

The EO compositions of various samples analyzed through GC-MS-FID are presented in Tables 2, 3 and 6. Overall, 56 compounds of EO were found, representing 98.8–99.9% of total EO (S2 Table). Data analyses of EOs compositions revealed three different classes of compounds, namely class monoterpenes (monoterpene hydrocarbons, oxygenated monoterpenes), class sesquiterpenes (sesquiterpenes hydrocarbons, and oxygenated sesquiterpenes), and aliphatic and aromatic hydrocarbons. The prevailing class of the EOs constituents were the monoterpenes (53.73–62.72%), followed by the sesquiterpenes (34.81–43.09%) (Table 7). *α*-Pinene (8.85–35.94%), limonene (11.81–50.08%), and cedrol (3.41–34.29%) were the most predominant compounds of EOs that were found in all analyzed samples, while *trans*-2,4-decadienol and *β*-caryophyllene were predominant in some individual trees (Table 3; S2 Fig). The statistical analysis showed significant variations in *α*-рinene, limonene, and cedrol between populations and between trees in the same population. For example, the samples from the reserve “Tisata” (East Pirin, location 1, Malesjevska Mountain, location 2), which are under the influence of a Continental-Mediterranean climate, on a silicate bedrock, *α*-рinene was 13.90% in Tree 6, and 33.96% in Tree 1 (Table 3). Similar variations of *α*-рinene were also found for the rest of the samples from the population in reserve “Tisata”, as well as for the samples from the population “Izgoryaloto Gyune” (Krichim) (Table 3). Likewise, limonene varied between populations and between individual trees. For example, high concentrations of limonene were detected in samples from the reserve “Tisata” (33.91%, Tree 5, location 1; 35.94%, Tree 10, location 2), as well as in samples from the reserve “Izgoryaloto Gyune” (Krichim) (50.08%, Tree 4; 45.20%, Tree 7), respectively (Table 3). In addition to the high concentrations of *α*-pinene and limonene, the EO *J*. *excelsa* in this study contained significant concentrations of cedrol (3.41–33.0%) (Table 3). The amount of cedrol varied between trees within the same population and between the two populations (Table 3). These variations of cedrol can be seen in the samples that were sampled from the reserve “Tisata” where cedrol was 3.41% (Tree 10) to 33.0% (Tree 2) Malesjevska mountain part (location 2), and 16.18% (Tree 5) to 29.25% (Tree 4) in East Pirin part (location 1) of the same reserve. A similar range of cedrol was observed in samples from the second studied population reserve “Izgoryaloto Gyune” (Krichim) (Table 3).

**Table 6:** Mean concentration (%) of intrapopulation variability of *β*-cedrene, *δ*-cadinene, caryophyllene oxide, 1,10-di-*epi* cubenol, *allo*-cedrol, and cubenol of *Juniperus excelsa* in Bulgaria obtained from 28 trees collected at the 3 locations (East Pirin, Krichim, and Malesjevska). e-i = efghi. Within each column, means sharing the same letter are not significantly different.

Tree	*β*-Cedrene	*δ*-Cadinene	Caryophyllene oxide	*allo*-Cedrol	1,10-di-epi Cubenol	Cubenol
**Population 1, Location 1**						
Tree1(EPT)	0.22 mn	1.75 b	0.25 lmn	1.36 i	0.94 e	0.43 mn
Tree2(EPT)	1.73 abc	1.51 c	1.75 b	1.99 c-h	1.41 b	1.57 cd
Tree3(EPT)	1.12 ghi	0.76 hij	1.14 d	1.53 hi	0.67 g-k	2.13 b
Tree4(EPT)	1.16 e-i	0.79 g-j	1.04 d	2.29 bcd	0.89 ef	2.72 a
Tree5(EPT)	1.55 cd	1.04 def	1.01 de	6.12 a	0.75 e-j	2.06 b
Tree6(EPT)	1.42 def	1.01 d-g	1.16 d	1.77 e-i	0.70 f-k	1.52 cde
**Population 1, Location 2**						
Tree1(MMT)	1.12 ghi	0.83 f-j	0.20 mn	1.70 f-i	0.83 e-h	0.33 n
Tree2(MMT)	1.75 abc	0.88 e-i	0.29 k-n	2.17 cde	1.18 cd	0.59 k-n
Tree3(MMT)	1.15 f-i	0.21 k	0.16 n	1.69 f-i	1.16 d	0.51 lmn
Tree4(MMT)	1.42 de	0.88 e-i	1.03 d	1.89 d-h	0.55 j-m	1.59 c
Tree5(MMT)	1.94 ab	1.83 ab	0.19 mn	2.13 c-f	1.39 bc	0.59 k-n
Tree6(MMT)	1.15 e-i	0.83 f-j	0.18 mn	2.13 c-f	1.89 a	0.73 jkl
Tree7(MMT)	1.58 bcd	1.99 a	2.30 a	2.01 c-h	1.27 bcd	0.53 lmn
Tree8(MMT)	1.57 bcd	0.93 e-h	0.62 f-i	1.67 ghi	0.59 i-m	1.46 c-f
Tree9(MMT)	1.26 e-h	0.85 e-i	0.49 h-k	2.02 c-g	0.59 i-m	0.87 ijk
Tree10(MMT)	1.83 ab	1.02 d-g	0.59 g-j	1.70 f-i	1.86 a	2.55 a
Tree11(MMT)	1.36 d-g	0.92 e-h	0.21 mn	2.17 cde	0.42 mn	1.26 e-h
Tree12(MMT)	2.69 a	0.98 ij	0.14 n	2.34 bc	0.20 n	0.31 n
**Population 2, Location 3**						
Tree1(IG)	0.78 jk	0.66 ij	0.44 i-l	2.13 c-f	0.64 h-l	1.40 c-f
Tree2(IG)	1.06 hi	0.82 f-j	0.82 ef	1.78 e-i	0.84 e-h	1.43 c-f
Tree3(IG)	1.05 hi	0.73 hij	1.41 c	2.02 c-g	0.78 e-i	0.72 j-m
Tree4(IG)	0.19 n	0.76 hij	0.65 fgh	1.32 i	0.45 lm	1.05 ghi
Tree5(IG)	0.49 lm	1.08 de	0.39 j-m	1.76 e-i	0.66 g-l	1.49 cde
Tree6(IG)	0.24 mn	0.66 ij	0.99 de	1.65 ghi	0.87 efg	1.28 d-g
Tree7(IG)	0.17 n	0.83 f-j	0.76 fg	1.59 ghi	0.67 g-k	1.18 fgh
Tree8(IG)	1.05 hi	1.18 d	0.58 g-j	1.28 i	0.69 f-k	1.29 c-g
Tree9(IG)	0.18 n	0.91 e-h	1.63 b	2.55 b	0.83 e-h	0.97 hij
Tree10(IG)	0.58 kl	0.66 ij	0.13 n	1.342 i	0.49 klm	0.54 lmn

EPT—East Pirin, Tisata; IG—Izgoryaloto Gyune; MMT—Malesjevska Mountain, Tisata.

**Table 7:** Mean concentration (%) of aliphatic hydrocarbons, aromatic hydrocarbons, monoterpene hydrocarbons, oxygenated monoterpenes, sesquiterpenes hydrocarbons, and oxygenated sesquiterpenes of *Juniperus excelsa* in Bulgaria obtained from the 3 locations (EastPirin, Krichim, and Malesjevska) (intrapopulation variability). Within each column, means sharing the same letter are not significantly different.

Location	Aliphatic hydrocarbons	Monoterpene hydrocarbons	Oxygenated monoterpenes	Sesquiterpene hydrocarbons	Oxygenated sesquiterpenes	Aromatic hydrocarbons,
EPT	2.79 a	50.22 b	3.51	10.66 a	32.43 a	0.19
IG	1.87 b	58.91 a	3.81	7.70 b	27.11 b	0.22
MMT	1.14 b	52.13 b	4.02	11.00 a	31.30 a	0.20

EPT—East Pirin, Tisata; IG—Izgoryaloto Gyune; MMT—Malesjevska Mountain, Tisata.

Other notable compounds of the EO were *trans*-2,4 decadienol (0.32–4.51%), and *β*-caryophyllene (1.73–4.76%), which were found in concentrations > 4% in the EO of some of the samples (Table 3). In this study, *β*-cedrene, *δ*-cadinene, caryophyllene oxide, *allo*-cedrol, 1,10-di-*epi* cubenol and cubenol of *J*. *excelsa* EO were found in quantities below 3% (Table 6).

#### 3.2.2. Seasonal variability of composition of EO

**Fig 2:**
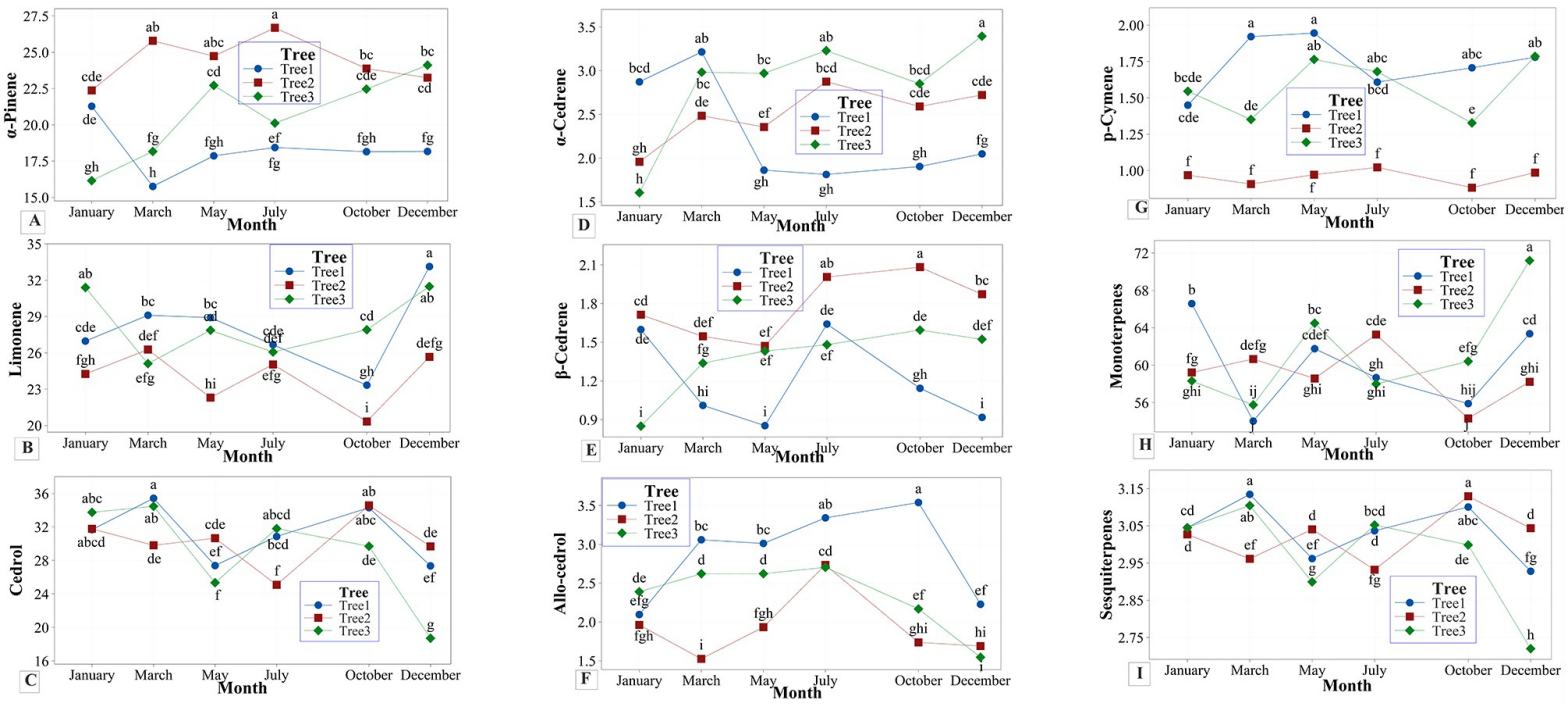
Interaction effect of Month and Tree on *α*-pinene [A], limonene [B], cedrol [C], *α*-cedrene [D], *β*-cedrene [E], *allo*-cedrol [F], *p*-cymene [G], monoterpenes [H], and sesquiterpenes [I] of *Juniperus excelsa* in Bulgaria obtained from 3 trees collected at 6 different months.

Seasonal variation of *J*. *excelsa* EOs is presented in Table 5 and Fig 2. GC-MS-FID analysis revealed quantitative and qualitative differences, both between trees and seasons. Overall, 35 EO constituents were detected (S3 Table). The main compounds of EO were *α*-pinene, limonene and cedrol, where their concentrations varied significantly between trees and between sampling times within a tree (Table 5; Fig 2). For example, *α*-pinene in Tree 1 was higher in January (21.2%) (Fig 2a) but its concentrations were not different between samples collected in other months (Table 5). The highest concentration of *α*-pinene for Tree 2 was in July (26.68%), but in Tree 3 the high amount of *α*-pinene is in December (24.11%) (Table 5). Overall, in this study, limonene was high in December for Tree 1 (33.13%) and Tree 3 (31.49%), but in Tree 2, the highest concentration was found in March (26.27%) (Table 5; Fig 2b). The concentration of cedrol in Tree 1 was the highest in March but in Tree 3 the highest was in October (Table 5; Fig 2c). Other EO constituents also varied significantly without a clear trend (Fig 2).

### 3.3. Concrete and resinoid

The results of the composition of the two aromatic products (concrete and resinoid) are presented in Table 8. A total of 28 concrete and resinoid compounds of *J*. *excelsa* were analyzed (S4 Table). The composition of concrete and resinoid was somewhat similar, but significant differences were also found. The main compounds of concrete were cedrol (15.39%), 7-hydroxy-4-methyl-coumarin (17.63%), 1-octacosanol respectively (36.85%), tritriacontane (16.08%), and tiacontanoic acid (4.79%) (Table 8). Cedrol, 7-hydroxy-4-methyl-coumarin, and 1-octacosanol were the predominant components of resinoids, respectively (Table 8). It was interesting to compare the composition of concrete and resinoid with the composition of the EO of the species. Concrete and resinoids were extracted from the samples collected from Tree 10 from Malesjevska Mountain. As seen in Table 4, *α*-pinene (35.94%), limonene (24.16%), and *β*-caryophyllene (4.66%) were the predominant compounds of EO of Tree 10, while cedrol was in a low amount (3.41%) (Table 3). Indeed, cedrol was one of the major compounds of concrete and resinoid found at concentrations 15.39% and 28.15%, respectively. Furthermore, 1-octacosanol (36.85%) and tritriacontane (16.08%) were also in high amounts, especially in concrete (Table 8).

**Table 8:** Compositions of concrete and resinoid of *Juniperus excelsa*.

Compounds	*Juniperus excelsa*		Compounds	*Juniperus excelsa*	
	Concrete	Resinoid		Concrete	Resinoid
(*E*)-Verbenol	0.13	nd	7-hydroxy-4,8-dimethyl-Coumarine	0.66	1.41
2*E*,4*E*-Decadienol	0.28	0.98	*n*-Heneicosane	nd	1.96
***β***-Cedrene	0.58	1.24	Abienol	0.27	1.26
Cubebol	0.64	0.34	dehydro-Abietal	0.20	0.75
(*E*)-Calamenene	0.21	0.41	4-epi-Abietal	0.24	2.14
Lauric acid	0.20	0.88	4-epi-Abietol	0.47	1.63
***Allo***-cedrol	1.04	1.82	dehydro-Abietol	0.53	1.89
Cedrol	15.39	28.15	Abietol	0.32	3.84
***epi***-Cedrol	0.27	0.62	Hexacosane	nd	2.61
5-Cedranone	1.00	1.71	1-Octacosanol	36.85	6.32
Junicedranone	0.24	1.27	Dotriacontane	nd	1.07
4-hydroxy-Coumarin	nd	5.83	Tritriacontane	16.08	nd
7-hydroxy-Coumarin	nd	3.10	Tiacontanoic acid (Melissic acid)	4.79	nd
7-hydroxy-4-methyl-Coumarin	17.63	26.94	Tetratriacontane	1.41	1.01

nd–not detected;

### 3.4. The test for herbicidal effect of EO against weed seeds of *Papaver rhoeas*, *Consolida orientalis*, *Anthemis arvensis*, *Avena fatua*, *Agrostemma githago*

**Fig 3:**
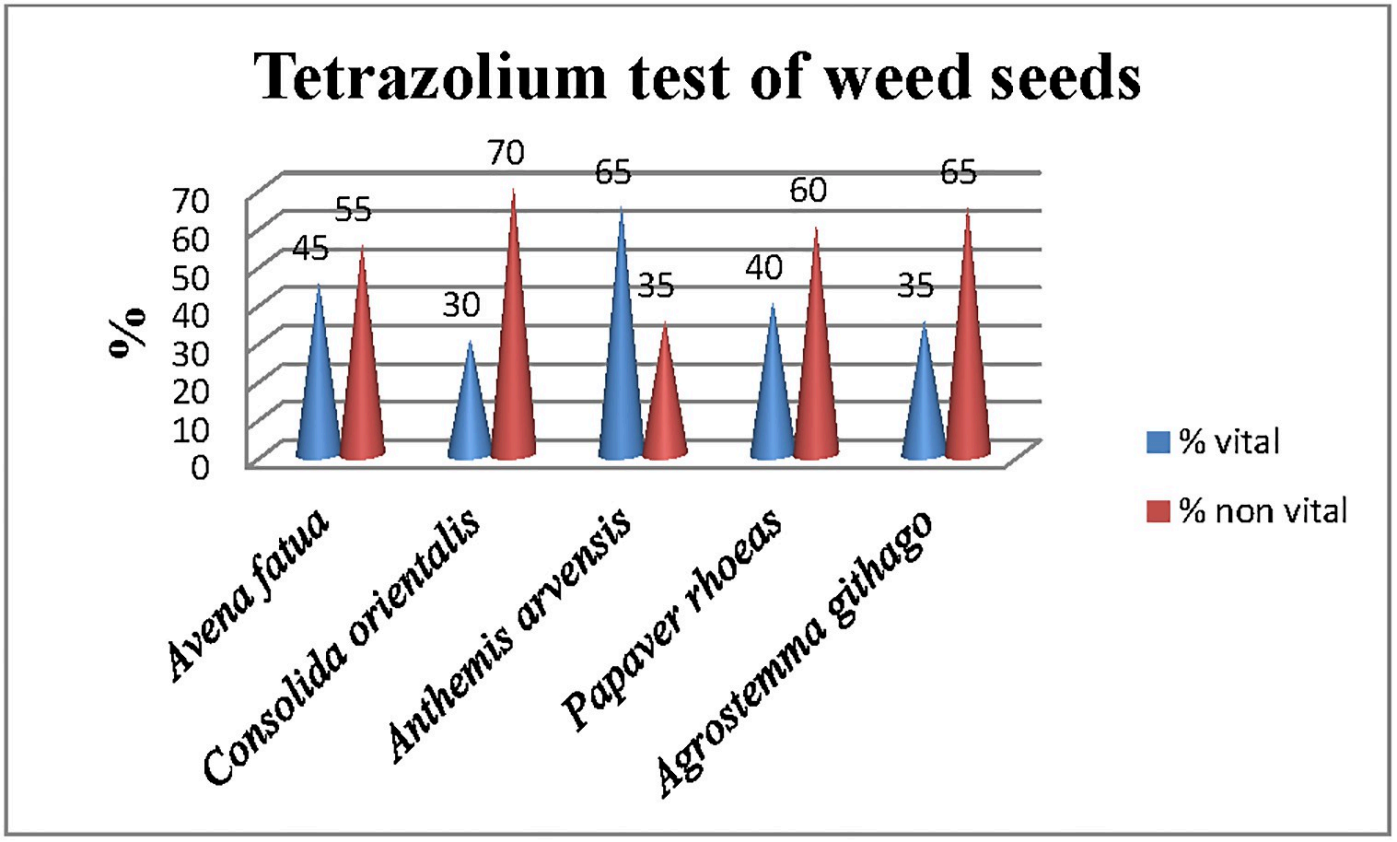
Tetrazolium test of weed seeds of *Papaver rhoeas*, *Consolida orientalis*, *Anthemis arvensis*, *Avena fatua*, *Agrostemma githago*.

In this study, potential herbicidal effect of *J*. *excelsa* EO was tested at 0 μL (control), 5 μL, 10 μL, and 20 μL concentrations in three replicates. According to the guidelines for laboratory studies on germination ecology [44], before conducting the tests for potential herbicidal effect, we did a test for imbibition water of weed seeds of *A*. *fatua*, *C*. *orientalis*, *P*. *rhoeas*, and *A*. *githago*. Mature seeds of some species do not germinate because seed coats are impermeable to water or embryos are non-viable [44]. To be sure that the seeds do not have physical and morphological dormancy, we performed tests for im bibition water and TT test (Table 9; Fig 3).

**Table 9:** Imbibition water of weed seeds of *Avena fatua*, *Consolida orientalis*, *Papaver rhoeas*, *Agrostemma githago*.

Species	Number seeds	Mean of weight of dry seeds in gram	Mean of weight of seeds in gram after imbibition of water	
		Mean±SD	Mean ±SD after 60 min	Mean ±SD after 120 min
*Avena fatua*	20	0.45±0.02	0.61±0.06	0.66±0.04
*Consolida orientalis*	20	0.10±0.01	0.14±0.00	0.17±0.01
*Papaver rhoeas*	50	0.04±0.00	0.05±0.00	0.05±0.00
*Agrostemma githago*	10	0.12±0.02	0.14±0.02	0.15±0.02

SD–standart deviation.

As shown in Table 9, all tested seeds imbibed water after the first 60 minutes of the test and water absorption continued in the following minutes of the test. So, these results showed that tested seeds had no physical dormancy. The TT test was conducted for all tested weed seeds (Fig 3). The range of the viability of seeds was from 30% (*C*. *orientalis*) to 65% (*A*. *arvensis*) (Fig 3). According to the official association of the Tetrazolium Subcommittee of the Association of Official Seed Analysts [45], the TT test indicated the viability of embryos in seeds that can be capable of producing normal plants under appropriate germination conditions.

In this study, the potential herbicidal effects of *J*. *excelsa* EO on germination, germination energy, sprout length, and root length of weed seeds were investigated in Petri dishes experiments. The seeds of *P*. *rhoeas*, *C*. *orientalis*, *A*. *arvensis*, *A*. *fatua*, and *A*. *githago* were tested because their management is difficult in fields. The results are presented in Table 10. Overall, germination and germination energy of *P*. *rhoeas* (16.5%), *C*. *orientalis* (15.0%), *A*. *fatua* (13.5%), and *A*. *githago* (26.5%) were lower than germination and germination energy of *A*. *arvensis* (68.5%) (Table 10). These lower germination results are related to the lower viability of target seeds (Fig 3). Overall, the highest germination energy (%) and germination were achieved in the Control seeds (Table 10). It is important to point out that the germination energy and germinated seeds were the same indicating that only initially germinated seeds develop sprouts.

**Table 10:** Average germination energy, germination, sprout length, and root length for *Papaver rhoeas*, *Consolida orientalis*, *Anthemis arvensis*, *Avena fatua*, *Agrostemma githago*.

Treatment in μL	Germination energy (%) ± SD	Germination (%) ± SD	Sprout length (cm) ± SD	Root length (cm) ± SD
	*Papaver rhoeas*			
Control (0 μL)	16.65 ± 0.57	16.65 ± 0.57	0.0	0.54 ± 0.02
*J*. *excelsa* 5μL	0.0	0.0	0.0	0.0
*J*. *excelsa* 10μL	0.0	0.0	0.0	0.0
*J*. *excelsa* 20μL	0.0	0.0	0.0	0.0
	*Consolida orientalis*			
Control (0 μL)	15.0 ± 1.0	15.0 ± 1.0	1.27 ± 0.10	0.97 ± 0.08
*J*. *excelsa* 5μL	0.0	0.0	0.0	0.0
*J*. *excelsa* 10μL	0.0	0.0	0.0	0.0
*J*. *excelsa* 20μL	0.0	0.0	0.0	0.0
	*Anthemis arvensis*			
Control (0 μL)	68.5 ±5.13	68.5 ± 5.13	1.26 ± 0.03	1.65 ± 0.05
*J*. *excelsa* 5μL	53.5 ± 3.51	53.5 ± 3.51	1.43 ± 0.11	1.44 ± 0.17
*J*. *excelsa* 10μL	18.5 ± 1.53	23.5 ± 1.15	0.24 ± 0.01	0.35 ± 0.02
*J*. *excelsa* 20μL	0.0 ± 0.0	11.5 ± 0.58	0.2 ± 0.13	0.32 ± 0.11
	*Avena fatua*			
Control (0 μL)	13.5 ± 0.58	13.5 ± 0.58	0.27 ± 0.46	0.9 ± 0.95
*J*. *excelsa* 5μL	0.0	0.0	0.0	0.0
*J*. *excelsa* 10μL	0.0	0.0	0.0	0.0
*J*. *excelsa* 20μL	0.0	0.0	0.0	0.0

SD–standart deviation.

The essential oil (EO) was found to be highly phytotoxic to the germination and seedling growth of *A*. *fatua*, *C*. *orientalis*, *P*. *rhoeas*, and *A*. *githago*, at all concentrations tested (5 μL, 10 μL, and 20 μL), as shown in Table 10. Consequently, no germination, germination energy, sprout length, or root lengths were observed for these species, as all values were recorded as zero (Table 10). The results also indicated a decrease in the percentage of germination energy and germination with an increase in the application rate of EO, as demonstrated in Table 10. For instance, the concentration of EO at 5 μL inhibited the germination of *A*. *arvensis* seeds up to 53.5%, while the concentration of EO at 10 μL reduced it up to 18.5%. At a concentration of 20 μL, the EO was highly phytotoxic (Table 10). Comparing the results with the control (68.5%), it was evident that the application of 5 μL of EO reduced germination by 21.9%, and the application of 10 μL reduced it by 73%. Moreover, an increase in EO concentration resulted in a reduction of sprout and root lengths from 1.43–1.44 cm (at 5 μL) to 0.24–0.35 cm (at 10 μL). In comparison, the germination rate in the control (0 μL) was 68.5%, and the sprout and root lengths were recorded as 1.26–1.65 cm (Table 10).

## 4. Discussion

### 4.1. Essential oil (EO) yield

*Juniperus excelsa* is known for relatively high amounts of EOs in berries (female cones) and leaves. This study observed a large variation in EO yields of *J*. *excelsa* leaves. These differences were results of the influence of many factors such as physiological variations, organ development, geographic and genetic factors, harvesting period, seasonal variation, method of extraction etc. [5, 51–53]. This study has been conducted in two isolated geographically (allopatric) populations of *J*. *excelsa*, and it found considerable variations in EO yield among populations and between individual trees within a population. As was noted in the results part, in some of the samples collected from Eastern Pirin, “Tisata”, the EO yield was 0.93%, while in other trees, the EO yield was 2.04%. Likewise, different yields were obtained from samples collected in the western part of the reserve “Tisata” (Malesevska Mountain) and the reserve “Izgoryaloto Gyune”, Krichim. Overall, our results on EO yield were in agreement with previous studies on species distributed in Iran [15, 37], Macedonia [8, 28], Lebanon [34], and Bulgaria [5]. Hojjati et al. [15] noted that the EO yield of species from 10 populations in Iran varied significantly from 0.08 to 3.78% dry weight (v/w). Our results did not show a trend in the EO yield linked to the climatic characteristics of the habitats. For example, we observed high yield values in both populations (at the three locations) that are under the influence of a Continental-Mediterranean climate (the reserve “Tisata”) and under the influence of a Moderate-Continental climate (the reserve “Izgoryaloto Gune”, Krichim) [54]. Similar variations in EO yield were observed for other species collected from different populations across Bulgaria as *J*. *oxycedrus* [53], *Satureja pilosa* Velen. and *Satureja kitaibelii* Wierzb. [53, 55], and *J*. *pygmaea* C. Koch., *J*. *sibirica* Burgsd., *J*. *communis* L. [56]. The main factors that influence the EO yield are mostly the individual trees’ genetic characteristics and the individual metabolism.

The results from the seasonal dynamics of EO study reported here support our conclusion. Overall, a wide range of variation in the yield of EO was found in samples collected from different seasons. For example, (1) Tree 1—the EO yield was significantly higher in samples collected in January (1.16%); (2) Tree 2—high EO yield was obtained in January and in December (0.75–0.72%); (3) Tree 3—the EO yield was high in samples collected in March (1.16%). Furthermore, we should note that the EO yield of Tree 1 and Tree 3 was almost two times higher than the EO yield of Tree 2. It seems, mostly the individual physiological and genetic traits determine the EO yield, because the studied trees are at a distance of one meter from each other, in the same conditions and soils. The variations of EO yield are well demonstrated in Fig 2, Tables 4 and 5. According to the results from this study, the EO yield depends first on the genetic characteristics of trees and then on the harvesting season. The trend of high EO yield in January and March contrast with the reported results for leaves’ EO of *J*. *excelsa* collected in Iran, where yield varied from spring (0.6%), summer (0.5%) to autumn (0.85%) [31]. Furthermore, the latter authors found that the galbuli EO yield increased by 162% from spring to autumn [31]. However, we should note that a total of three samples from 3 trees in April, August and November for the entire year were examined in their study, and the results of the three trees were merged [31]. Therefore, their study did not show clear seasonal dynamics of EO yields. Generally, our data showed that the best period of harvesting *J*. *excelsa* samples with a high EO yield was in January, or December, while in May, July, and October, the EO yield was low.

### 4.2. Essential Oil (EO) compositions of *Juniperus excelsa*

#### 4.2.1. Intrapopulation and interpopulation composition of EO

Generally, in the studied samples of *J*. *excelsa* collected from Bulgaria, *α*-pinene (8.85–35.94%), limonene (11.81–50.08%), and cedrol (3.41–34.29%) were the most predominant EO compounds. Most of these compounds (*α*-pinene, limonene, cedrol) were reported previously for Bulgarian samples of this species [5]. As shown in Table 1, limonene was reported in samples from Greece [27], Azerbaijan and Iran [18], Turkey [29, 30], but it was not detected in most of the samples of other studies [17, 31]. *α*-Pinene, limonene and cedrol were found in all analyzed samples in varying amounts, while *trans*-2,4-decadienol and *β*-caryophyllene were predominant for individual trees. The statistical analysis showed significant variations in the quantitative parameters of all compounds between populations and between trees in the same population. When comparing published data with the results from this study, both similarities as well as many differences are obvious. Some authors cited in Table 1 found *α*-pinene as the prevailing component of *J*. *excelsa* leaf EO (Table 1), but the quantities were variable. For example, *α*-pinene reported in samples from Iran ranged from 67.71 to 73.27% [14, 37], from R. Macedonia it was 33.88% [8], from Azerbaijan/Iran 32.75–59.90% [18], from Turkey 55.5% [9]. A previously report from Bulgarian samples showed that of *α*-pinene range from 18.90 to 22.30% [5].

**Fig 4:**
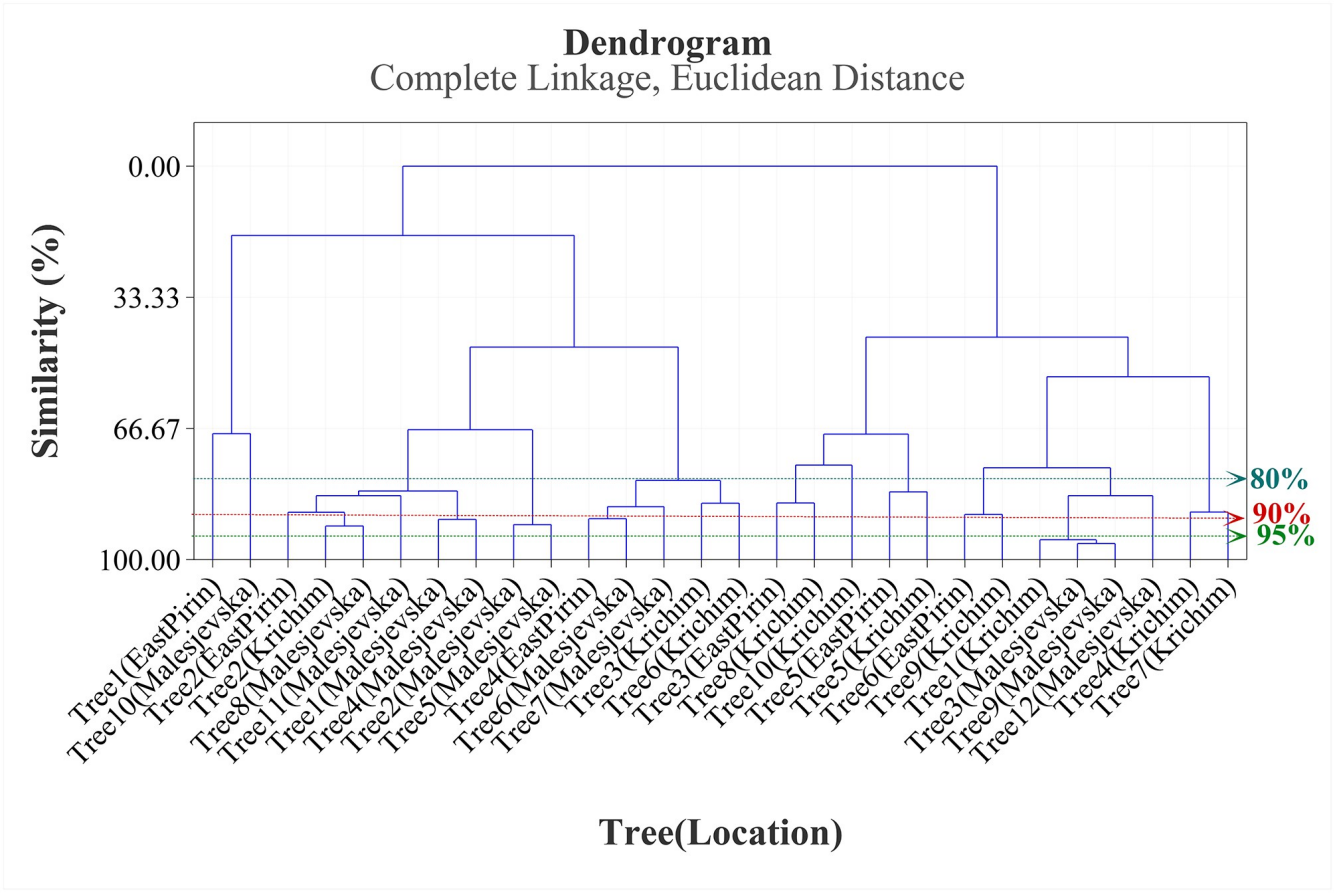
Complete linkage dendrogram showing the similarity of 28 combinations of Tree and Location in terms of *α*-pinene, limonene, *trans*-2,4-decadienol, *β*-caryophyllene, *β*-cedrene, *δ*-cadinene, caryophyllene oxide, *allo*-cedrol, cedrol, 1,10-di-*epi*-cubenol, and cubenol.

Generally, the phytochemical diversity of *J*. *excelsа* trees distributed in Bulgarian flora was well presented in Fig 4. The complete linkage Dendrogram (Fig 4) showed the similarity level of the locations in terms of all 11 constituents shown in Table 4. The cluster analysis showed 90% similarity of samples of reserve “Izgoryaloto Gyune” (Krichim) as well as samples from two locations of reserve “Tisata” (East Pirin, Malesjevska Mountain). According to the statistical results and percent ratio of the predominant compounds of *J*. *excelsa* EO, trees can be clustered into four chemical groups as follows: Type (1) *α*-pinene, limonene, cedrol; Type (2) *α*-pinene, limonene, *trans*-2,4-decadienol; Type (3) *α*-pinene, limonene, *β*-caryophyllene; and Type (4) *α*-pinene, limonene, *β*-caryophyllene, cedrol. The chemical groups were determined based on the lowest concentration as % of the total oil (>4%) of the predominant components.

Similar to our 1^st^ chemical groups compositions of *J*. *excelsa* EO were reported by Adams [27] for samples from Greece (*α*-pinene 22.5%, cedrol 28.1%, limonene 22.7%), Topçu et al. [29] from Turkey (*α*-pinene 29.7%, cedrol 25.3%), and Asili et al. [30] from Iran (*α*-pinene 32.34%, cedrol 13.06%) (Table 1). Somewhat controversial results of the composition of EO were reported by Sela et al. [8] for samples from R. Macedonia. The cited authors reported two chemotypes in this species based on the EO composition; (1) *α*-pinene-type, containing *α*-pinene, limonene, *β*-pinene, and *β*-myrcene, and (2) sabinene-type, containing mainly sabinene followed by *α*-pinene, *β*-myrcene, limonene, *cis*-thujone, terpinolene, and *α*-thujene [8]. In another study, sabinene (72.80%) and myrcene (5.56%) were reported as the main compounds of *J*. *excelsa* EO [28]. In our study, sabinene was not found and the concentrations of terpinolene, myrcene, and *β*-pinene were low (<1.0%) (S2 Table).

It is important to note that the results obtained in this study of *J*. *excelsa* EO cannot be linked to the geographical location of the populations (Fig 4). Likewise, no clear trend between the composition of EO and the ecological conditions was observed (Fig 4). Apparently, in past geological epochs, the species had a wider distribution in Bulgaria, and probably the populations of *J*. *excelsa* were sympatric. Because of the relatedness of populations between species, there had been a free exchange of genetic material within populations. This was a possible cause of the phytochemical diversity of *J*. *excelsa*. Due to the tectonic movements of the earth’s crust, the earth’s surface was repeatedly glaciated and changed [57]. Only the lowest parts of the earth’s surface were unglaciated, and it was refuge in which species survived. As a consequence of changes in the earth’s surface, populations are isolated geographically (allopatric). To establish the diversity of Bulgarian populations of *J*. *excelsa*, broader phytochemical and genetic studies are needed.

#### 4.2.2. Seasonal variability of composition of EO

Overall, 35 constituents of EO were detected in seasonal compositions of EOs (S3 Table). The main compounds of EO were *α*-pinene, limonene and cedrol. Their quantity varied significantly both between trees and between sampling times and it was clearly visible in Fig 2. For example, *α*-pinene in Tree 1 was higher in January, while for Tree 2 it was higher in July (Fig 2a). Our result was partly in contradiction to the result of Shanjani et al. [31]. The cited authors found that, *α*-pinene, *trans*-verbenol, and germacrene B decreased in summer but in our study *α*-pinene for Tree 2 was very high in July. Significant individual variations between trees and sample collection times were found for limonene, cedrol and other compounds of EOs (Fig 2). Limonene was higher in December for Tree 1 (33.13%), and Tree 3 (31.49%), while in Tree 2, limonene was higher in March (26.27%). Cedrol increased in March for Tree 1 (35.42%) and Tree 3 (34.47%). In contrast, cedrol in Tree 2 was higher in October (Fig 2c). Cedrol is an aromatic component valued for producing perfumes, and *J*. *excelsa* EO is known as a natural source of cedrol [58]. Therefore, trees with high cedrol content could be introduced species into the culture for cedrol production. The results from this study suggest that the individual genetic characteristics determine the differences in EO compositions.

Overall, the individual genetic traits and the sampling time significantly affect the EO composition of *J*. *excelsa*. Since the studied trees are only 1–2 meters apart from each other, they grow under the same ecological conditions. Also, the samples were collected in the same way and time, and the EO was extracted under the same conditions. Generally, our results show that the best period of harvesting *J*. *excelsa* samples for high EO yield and higher content of limonene and cedrol is during the winter months (December, January, or March).

### 4.3. Concrete and resinoid

Plant EOs, concretes and resinoids are widely used by the fragrance and cosmetics industries [59, 60]. The species from the genus *Juniperus* are known for their specific odors and they would offer potential for cosmetics industries [61]. As stated above, *J*. *excelsa* EO is rich in cedrol. It is an aromatic component valued for producing perfumes, and *J*. *excelsa* EO is a natural source of cedrol [58]. The composition of concrete and resinoid in this study was compared with *J*. *excelsa* EO. The composition of these aromatic products showed more differences than similarities with EO. For example, the EO composition of Tree 10 of which sample were made concrete and resinoid contains *α*-pinene (35.94%), limonene (24.16%), and *β*-caryophyllene (4.66%). Cedrol was at low amount (3.41%) of this Tree (Table 3). In contrast, in concrete cedrol was 15.39%, and in resinoid it was 28.15%, respectively. Furthermore, 7-hydroxy-4-methyl-coumarin, and 1-octacosanol were found only in concrete and resinoid. Besides, 1-octacosanol (36.85%) and tritriacontane (16.08%) were also in high amounts, especially in concrete (Table 8). The concrete and resinoid composition of *J*. *excelsa* should be cautionary interpreted since individual and geographical variations were not included in the present study. Indeed, 1-octacosanol, and tritriacontane are common compounds of epicuticle waxes of plants, and they are very hydrophobic, and insoluble in water. As mentioned, *J*. *excelsa* is distributed in sclerophyllous type habitats and extends along the steep slopes of deep, rocky places, exposed to high solar radiation. The formation of waxes is a defense mechanism of plants. Furthermore, octacosanol has been studied for its potential to treat Parkinson’s disease [62, 63]. An important objective for future research on the aromatic product of *J*. *excelsa* would be establishing new biological activities. The 7-hydroxy-4-methyl-coumarin was found in both the concrete (17.63%), and the resinoid (26.94%). This compound was reported to exhibit good fungicidal and insecticidal activities [64]. Generally, this is the first report on the compositions of *J*. *excelsa* concrete and resinoid.

### 4.4. Herbicidal effect of EO against weed seeds of *Papaver rhoeas*, *Consolida orientalis*, *Anthemis arvensis*, *Avena fatua*, *Agrostemma githago*

While synthetic herbicides are not allowed in certified organic production, the uses of biological methods are allowed. Because the EOs are less phytotoxic volatile, and friendly to the environment, they are often used for controlling weeds [65]. As a known *P*. *rhoeas*, *C*. *orientalis*, *A*. *arvensis*, *A*. *fatua*, and *A*. *githago* weeds cause considerable losses in organic field crops and EO is a good bioherbicides option for farmers. The herbicidal potential of various EOs is well documented [19, 24, 66]. Regarding the germination and seedling growth of the weeds of *A*. *fatua*, *C*. *orientalis*, *P*. *rhoeas*, and *A*. *githago*, the EO was highly phytotoxic on seed in all concentrations (5 μL, 10 μL, and 20 μL) (Table 10). Consequently, the results of germination, germination energy, sprout length, and root lengths of seeds for these species were zero (Table 10). Similar results of the inhibitions of seed germination of *Melilotus officinalis* L. were observed in our previous study [19], but the results were somewhat contradictory. For example, the *J*. *excelsa* EO (SCom) at 60 and 90 μL inhibited germination, but a concentration at 30 μL stimulated germination energy and increased germination to 100% [19]. Among the tested seeds, only seeds of *A*. *arvensis* showed high germination energy and germination (Table 10). It is well known that germination energy (%) is an important seeds parameter that indicates how many seeds would germinate simultaneously under optimal conditions [67]. Our results indicated that the percentage of germination energy and germination of seeds of *A*. *arvensis* decreased with increasing of the EO application rate. Similar results of strong potential herbicidal potential of EOs were reported by other authors [68–70]. Monoterpenes and sesquiterpenes are often reported to be the responsible compounds for the observed inhibitory activity on seeds germination [70], and *J*. *excelsa* EO is a rich of them. The exact phytotoxic mechanism by which germination energy, germination, sprout length, and root length are affected by *J*. *excelsa* EO is unknown. According to Kordali et al. [71] EO compounds influence receptors in the plasma membrane of the embryonic cells. Furthermore, they provoke the synthesis of phytohormones that are either stimulated or inhibit germination [71]. As was mentioned in Materials and methods part (2.3.3.), *J*. *excelsa* EO composition was previously reported, and *α*-cedrol (24.06–27.00%), *α*-pinene (18.90–22.30%), and *α*-limonene (23.23–27.50%) were the main compounds of EO extracted in semi-commercial distillation apparatus by steam distillation [5]. It has been known that *α*-pinene and limonene affect the respiration of cells and decrease energy in seeds [72, 73]. Since we have not tested pure substances in this study, we cannot determine the exact input of each ingredient of EO on the observed effects. According to reported data for limonene, *α*-pinene, camphene, *β*-pinene, borneol, pulegone, carvacrol, thymol, *p*-cymene, and 1,8-cineole, there were suggestions for their allelopathy potential [65, 74, 75]. The latter authors used whole EO, and as is well known, the EOs are mixes of compounds and it is difficult to determine which compounds exactly have an herbicidal effect. Furthermore, there are several studies that showed that the herbicidal activity of the EO may be due to synergetic and antagonistic interactions of various compounds, and interaction between nutrients in the seeds and water [71, 76, 77]. There are various assumptions about the mechanism of herbicidal action of EOs, but it needs future research.

## 5. Conclusion

The present study demonstrated the phytochemical variability of *J*. *excelsa* essential oil (EO) among trees within a population, between populations, and across different seasons. The results suggest that the yield and composition of the EO depend most probably on the genetic characteristics of the trees and then on the harvesting season. Based on the variations in EO composition, four chemical groups were identified: Type (1) *α*-pinene, limonene, cedrol; Type (2) *α*-pinene, limonene, *trans*-2,4-decadienol; Type (3) *α*-pinene, limonene, *β*-caryophyllene; and Type (4) *α*-pinene, limonene, *β*-caryophyllene, and cedrol. This is the first report on the existence of four chemotypes in *J*. *excelsa* in Bulgaria and in the world. The winter months were found to be the best period for harvesting *J*. *excelsa* samples to obtain high EO yield. In addition, this is the first report on the compositions of *J*. *excelsa* concrete and resinoid, which had different composition from that of the EOs, and may have potential for new applications. Furthermore, *J*. *excelsa* EO were found to be highly phytotoxic to the seeds of *P*. *rhoeas*, *C*. *orientalis*, *A*. *arvensis*, *A*. *fatua*, and *A*. *githago*. Thus, *J*. *excelsa* EO has a potential to be used in the development of new bioherbicides.

## Supporting information

S1 Fig UTM map of the collection sites of *Juniperus excelsa* in Bulgaria. (PDF)

S2 Fig Representative chromatograms of *Juniperus excelsa* concrete (A), resinoid (B), and essential oils (C) intrapopulation, (B) seasonal variation. (PDF)

S1 File (DOCX)

S1 Table Coordinates, meters above sea level (masl) in populations of Juniperus excelsa from Bulgaria. (PDF)

S2 Table Average composition of *Juniperus excelsa* EOs from two populations from Bulgaria. (PDF)

S3 Table Average composition of seasonal variability of *Juniperus excelsa* EOs. (PDF)

S4 Table Compounds of concrete and resinoid of *Juniperus excelsa*. (PDF)
