# Supplementary figures and images for: Chemical profile of Juniperus excelsa M. Bieb. essential oil within and between populations and its weed seed suppression effect
Source: PLoS One. 2024 Feb 8;19(2):e0294126. doi: 10.1371/journal.pone.0294126 (PMC10852245; doi:10.1371/journal.pone.0294126)

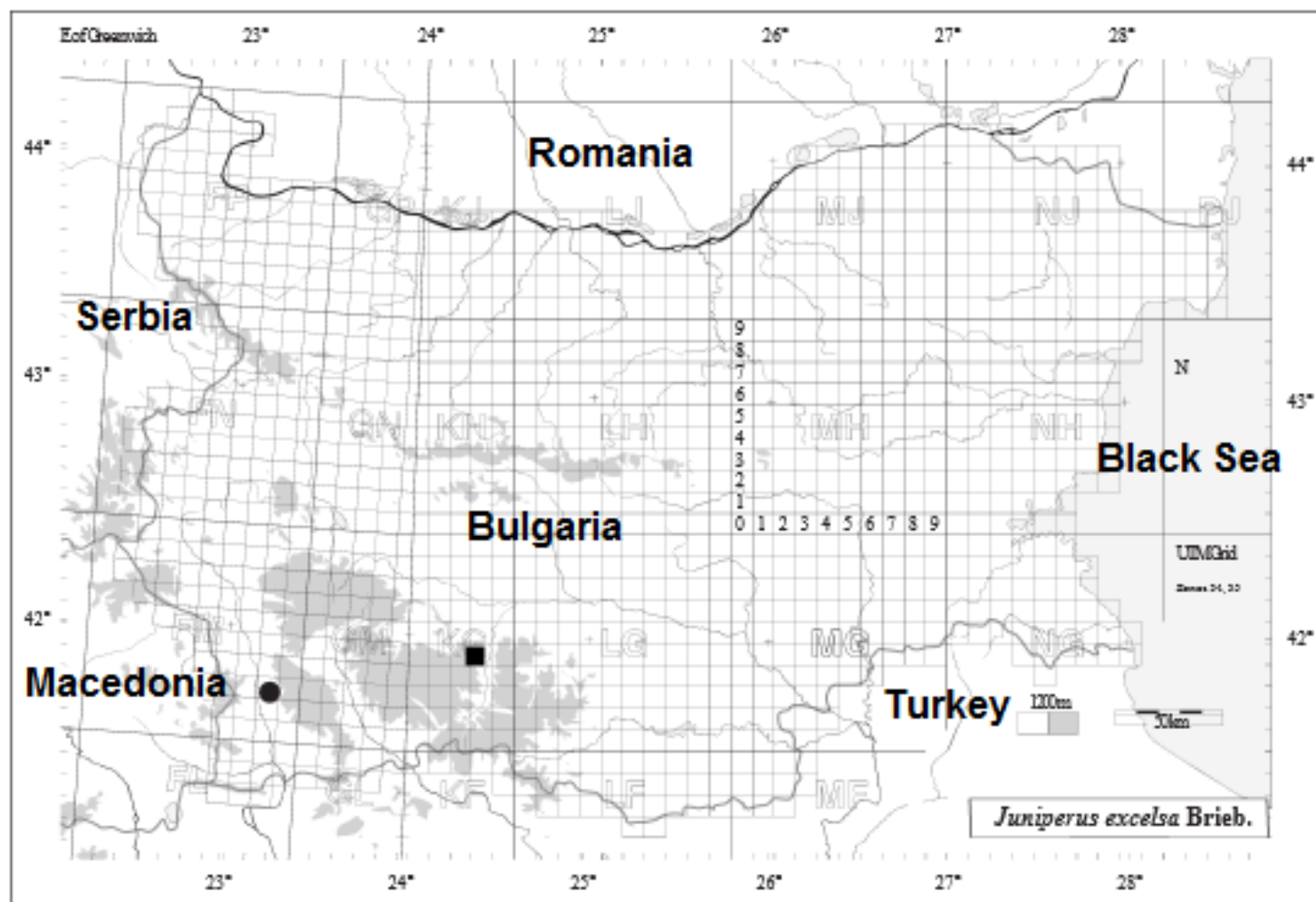

- The reserve "Izgoryaloto Gyune"
- The reserve "Tisata"

Supplement: S1 Fig — (PDF) [file pone.0294126.s001.pdf]
