# Supplementary material for: Chemical profile of Juniperus excelsa M. Bieb. essential oil within and between populations and its weed seed suppression effect
Source: PLoS One. 2024 Feb 8;19(2):e0294126. doi: 10.1371/journal.pone.0294126 (PMC10852245; doi:10.1371/journal.pone.0294126)
Supplement: S2 Fig — (PDF) [file pone.0294126.s002.pdf]

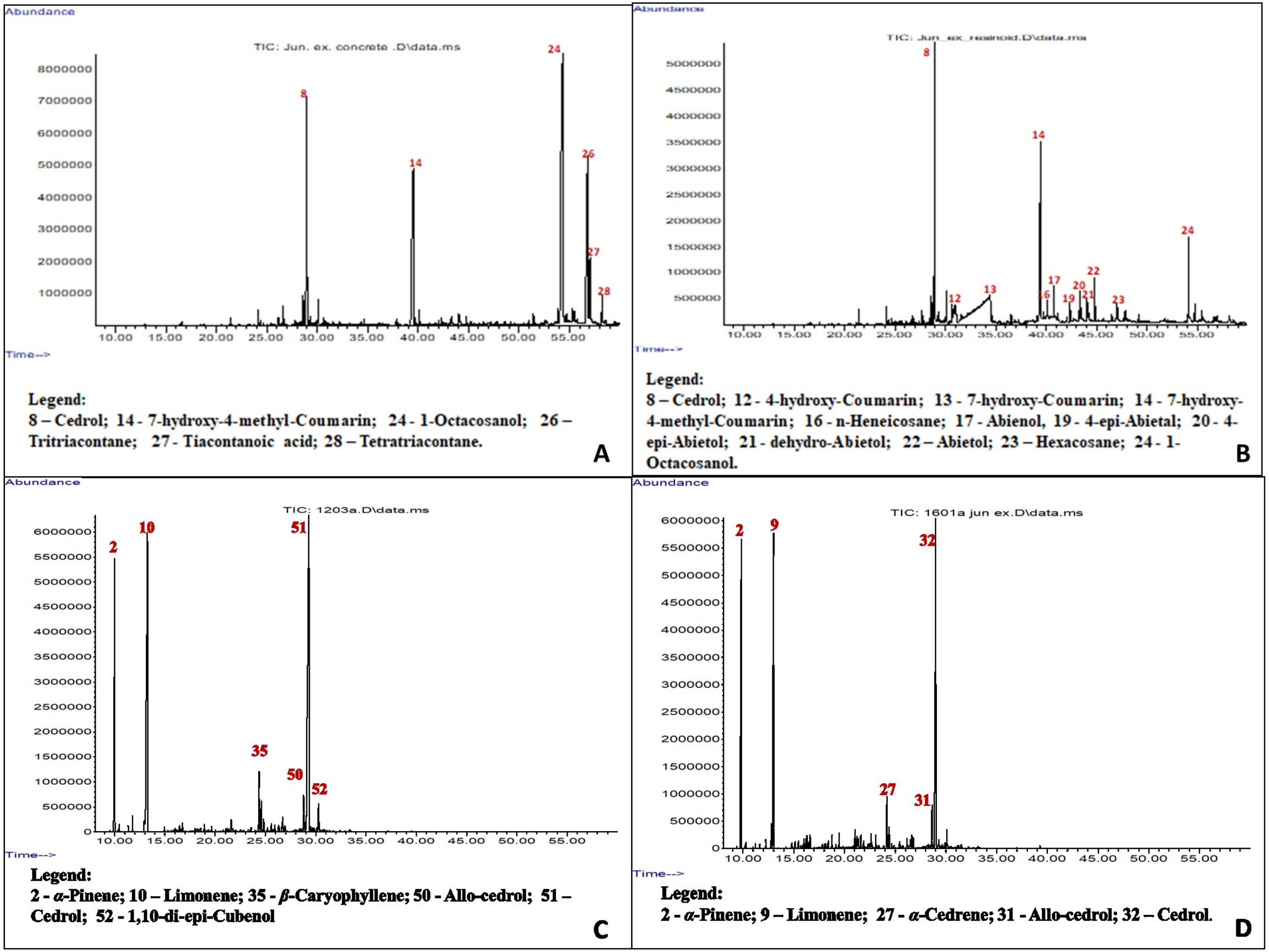
Abundance
TIC: Jun. ex. concrete .D\data.ms
Time-->
Legend:
8 – Cedrol; 14 - 7-hydroxy-4-methyl-Coumarin; 24 - 1-Octacosanol; 26 – Tritriacontane; 27 - Tiacontanoic acid; 28 – Tetratriacontane.
A
Abundance
Time-->
Legend:
8 – Cedrol; 12 - 4-hydroxy-Coumarin; 13 - 7-hydroxy-Coumarin; 14 - 7-hydroxy-4-methyl-Coumarin; 16 - n-Heneicosane; 17 - Abienol, 19 - 4-epi-Abietal; 20 - 4-epi-Abietol; 21 - dehydro-Abietol; 22 – Abietol; 23 – Hexacosane; 24 - 1-Octacosanol.
B
Abundance
TIC: 1203a.D\data.ms
Time-->
Legend:
2 - α-Pinene; 10 – Limonene; 35 - β-Caryophyllene; 50 - Allo-cedrol; 51 – Cedrol; 52 - 1,10-di-epi-Cubenol
C
Abundance
TIC: 1601a jun ex.D\data.ms
Time-->
Legend:
2 - α-Pinene; 9 – Limonene; 27 - α-Cedrene; 31 - Allo-cedrol; 32 – Cedrol.
D
