# Supplementary material for: Chemical profile of Juniperus excelsa M. Bieb. essential oil within and between populations and its weed seed suppression effect
Source: PLoS One. 2024 Feb 8;19(2):e0294126. doi: 10.1371/journal.pone.0294126 (PMC10852245; doi:10.1371/journal.pone.0294126)
Supplement: S1 File — (DOCX) [file pone.0294126.s003.docx]

**Table 1** Data used for the Nested Design statistical analysis.

| Location | Tree | Rep | Oil yield | α-Pinene | Limonene | trans-2,4-Decadienol | β-Caryophyllene | β-Cedrene | δ-Cadinene | Caryophyllene oxide | Allo-cedrol | Cedrol | 1,10-di-epi-Cubenol | Cubenol | Aliphatic hydrocarbons, % | Monoterpene hydrocarbons, % | Oxygenated monoterpenes, % | Sesquiterpene hydrocarbons, % | Oxygenated sesquiterpenes, % | Aromatic hydrocarbons, % | Oxigenated aromatics, % |
| --- | --- | --- | --- | --- | --- | --- | --- | --- | --- | --- | --- | --- | --- | --- | --- | --- | --- | --- | --- | --- | --- |
| A | Tree1 | 1 | 1.73 | 23.40 | 28.80 | 1.93 | 3.25 | 1.05 | 0.78 | 0.19 | 1.60 | 23.69 | 0.78 | 0.31 | 1.95 | 55.97 | 2.78 | 9.01 | 29.87 | 0.25 | 0.17 |
| A | Tree1 | 2 | 1.84 | 22.29 | 27.49 | 2.17 | 3.65 | 1.18 | 0.88 | 0.21 | 1.80 | 22.76 | 0.87 | 0.35 | 2.21 | 54.19 | 3.14 | 10.17 | 29.81 | 0.29 | 0.19 |
| A | Tree2 | 1 | 1.58 | 17.16 | 26.56 | 0.53 | 2.81 | 1.65 | 0.83 | 0.28 | 2.04 | 33.59 | 1.11 | 0.55 | 0.32 | 55.55 | 6.84 | 7.22 | 29.66 | 0.24 | 0.17 |
| A | Tree2 | 2 | 1.47 | 16.43 | 25.54 | 0.59 | 3.16 | 1.85 | 0.93 | 0.31 | 2.29 | 32.40 | 1.25 | 0.62 | 0.96 | 53.69 | 3.09 | 10.14 | 31.77 | 0.20 | 0.15 |
| A | Tree3 | 1 | 1.18 | 15.67 | 38.74 | 0.28 | 2.33 | 1.08 | 0.20 | 0.15 | 1.59 | 24.72 | 1.09 | 0.48 | 1.08 | 52.11 | 3.46 | 11.37 | 31.58 | 0.23 | 0.17 |
| A | Tree3 | 2 | 1.52 | 14.80 | 37.85 | 0.31 | 2.62 | 1.21 | 0.22 | 0.17 | 1.79 | 23.87 | 1.22 | 0.54 | 0.43 | 42.52 | 4.00 | 11.68 | 41.03 | 0.10 | 0.23 |
| 1A | Tree4 | 1 | 1.14 | 25.54 | 25.12 | 0.95 | 3.80 | 1.34 | 0.83 | 0.97 | 1.78 | 24.77 | 0.52 | 1.50 | 0.47 | 39.79 | 4.36 | 12.73 | 42.29 | 0.11 | 0.25 |
| A | Tree4 | 2 | 1.10 | 24.69 | 24.22 | 1.07 | 4.27 | 1.50 | 0.93 | 1.09 | 2.00 | 23.83 | 0.58 | 1.68 | 0.13 | 49.54 | 5.00 | 8.22 | 36.65 | 0.26 | 0.20 |
| A | Tree5 | 1 | 1.70 | 14.29 | 24.12 | 0.41 | 2.87 | 1.82 | 1.72 | 0.18 | 2.01 | 33.41 | 1.31 | 0.55 | 0.15 | 48.25 | 5.63 | 9.26 | 36.19 | 0.29 | 0.22 |
| A | Tree5 | 2 | 1.72 | 13.41 | 23.31 | 0.46 | 3.22 | 2.05 | 1.93 | 0.20 | 2.25 | 35.15 | 1.47 | 0.62 | 0.30 | 48.10 | 3.23 | 14.11 | 33.95 | 0.14 | 0.16 |
| A | Tree6 | 1 | 1.53 | 30.06 | 15.77 | 0.13 | 2.22 | 1.08 | 0.78 | 0.17 | 2.01 | 29.67 | 1.78 | 0.69 | 0.34 | 45.89 | 3.62 | 15.81 | 33.99 | 0.16 | 0.18 |
| A | Tree6 | 2 | 1.35 | 29.48 | 14.60 | 0.14 | 2.49 | 1.22 | 0.87 | 0.19 | 2.25 | 28.33 | 2.00 | 0.77 | 2.39 | 52.10 | 3.03 | 8.54 | 33.44 | 0.26 | 0.23 |
| A | Tree7 | 1 | 0.98 | 27.21 | 17.52 | 0.30 | 4.68 | 1.49 | 1.88 | 2.17 | 1.89 | 26.35 | 1.19 | 0.50 | 2.68 | 50.45 | 3.41 | 9.61 | 33.30 | 0.29 | 0.26 |
| A | Tree7 | 2 | 1.08 | 26.16 | 16.18 | 0.34 | 4.83 | 1.67 | 2.11 | 2.43 | 2.12 | 25.60 | 1.34 | 0.56 | 0.34 | 45.89 | 3.62 | 15.81 | 33.99 | 0.16 | 0.18 |
| A | Tree8 | 1 | 1.09 | 23.79 | 22.40 | 2.35 | 2.74 | 1.47 | 0.88 | 0.58 | 1.57 | 26.14 | 0.55 | 1.38 | 1.76 | 55.68 | 2.97 | 8.69 | 30.30 | 0.21 | 0.39 |
| A | Tree8 | 2 | 1.09 | 22.73 | 21.17 | 2.64 | 3.07 | 1.66 | 0.98 | 0.65 | 1.77 | 25.14 | 0.62 | 1.54 | 0.29 | 66.11 | 4.95 | 12.39 | 15.65 | 0.22 | 0.40 |
| A | Tree9 | 1 | 1.67 | 15.44 | 38.40 | 1.54 | 2.98 | 1.19 | 0.80 | 0.46 | 1.90 | 24.74 | 0.56 | 0.82 | 0.33 | 64.39 | 5.55 | 13.90 | 15.16 | 0.25 | 0.44 |
| A | Tree9 | 2 | 1.34 | 14.55 | 37.54 | 1.73 | 3.35 | 1.33 | 0.90 | 0.52 | 2.13 | 23.89 | 0.63 | 0.92 | 0.84 | 51.69 | 4.05 | 8.15 | 34.98 | 0.14 | 0.16 |
| A | Tree10 | 1 | 1.21 | 36.31 | 24.83 | 0.29 | 4.38 | 1.72 | 0.96 | 0.55 | 1.60 | 3.82 | 1.75 | 2.67 | 0.95 | 50.16 | 4.56 | 9.18 | 34.82 | 0.15 | 0.18 |
| A | Tree10 | 2 | 1.25 | 35.58 | 23.50 | 0.32 | 4.93 | 1.94 | 1.08 | 0.62 | 1.80 | 2.94 | 1.96 | 2.42 | 1.68 | 52.92 | 3.62 | 9.96 | 31.52 | 0.19 | 0.12 |
| A | Tree11 | 1 | 2.06 | 20.52 | 27.56 | 0.83 | 2.42 | 1.28 | 0.86 | 0.20 | 2.04 | 29.48 | 0.39 | 1.19 | 1.88 | 51.30 | 4.06 | 11.15 | 31.27 | 0.21 | 0.13 |
| A | Tree11 | 2 | 2.07 | 19.60 | 26.50 | 0.93 | 2.72 | 1.44 | 0.97 | 0.22 | 2.29 | 28.62 | 0.44 | 1.33 | 1.75 | 60.88 | 4.11 | 10.91 | 22.06 | 0.10 | 0.19 |
| A | Tree12 | 1 | 2.70 | 9.29 | 40.74 | 1.65 | 2.53 | 0.92 | 0.58 | 0.13 | 2.20 | 27.40 | 0.19 | 0.29 | 1.97 | 59.01 | 4.62 | 12.28 | 21.79 | 0.11 | 0.21 |
| A | Tree12 | 2 | 2.44 | 8.43 | 39.88 | 1.85 | 2.84 | 1.03 | 0.65 | 0.15 | 2.47 | 26.78 | 0.21 | 0.33 | 2.04 | 44.95 | 2.77 | 13.71 | 36.02 | 0.21 | 0.29 |
| B | Tree1 | 1 | 2.12 | 34.44 | 21.09 | 1.73 | 2.01 | 0.20 | 1.64 | 0.23 | 1.28 | 17.53 | 0.88 | 0.40 | 2.29 | 42.26 | 3.10 | 15.36 | 36.43 | 0.24 | 0.32 |
| B | Tree1 | 2 | 1.96 | 33.49 | 20.98 | 1.95 | 2.26 | 0.23 | 1.85 | 0.26 | 1.43 | 16.69 | 0.99 | 0.45 | 1.56 | 57.10 | 3.81 | 9.28 | 27.82 | 0.25 | 0.18 |
| B | Tree2 | 1 | 1.14 | 18.97 | 22.72 | 2.02 | 2.90 | 1.63 | 1.42 | 1.65 | 1.88 | 24.72 | 1.33 | 1.48 | 1.75 | 55.66 | 4.28 | 10.41 | 27.41 | 0.28 | 0.20 |
| B | Tree2 | 2 | 0.72 | 17.31 | 21.53 | 2.27 | 3.26 | 1.83 | 1.59 | 1.85 | 2.11 | 23.89 | 1.49 | 1.66 | 2.29 | 42.26 | 3.10 | 15.36 | 36.43 | 0.24 | 0.32 |
| B | Tree3 | 1 | 1.60 | 21.38 | 32.54 | 1.53 | 2.65 | 1.05 | 0.71 | 1.07 | 1.44 | 19.86 | 0.63 | 2.00 | 2.75 | 43.08 | 4.14 | 9.88 | 39.83 | 0.18 | 0.13 |
| B | Tree3 | 2 | 1.63 | 20.50 | 31.76 | 1.72 | 2.98 | 1.18 | 0.80 | 1.21 | 1.61 | 18.55 | 0.70 | 2.25 | 2.89 | 54.52 | 2.93 | 10.27 | 29.01 | 0.17 | 0.21 |
| B | Tree4 | 1 | 1.19 | 27.73 | 13.96 | 2.41 | 3.02 | 1.09 | 0.75 | 0.98 | 2.16 | 29.83 | 0.84 | 2.56 | 3.25 | 52.63 | 3.30 | 11.56 | 28.82 | 0.19 | 0.24 |
| B | Tree4 | 2 | 1.25 | 26.42 | 12.79 | 2.71 | 3.40 | 1.23 | 0.84 | 1.10 | 2.42 | 28.65 | 0.94 | 2.87 | 3.86 | 50.21 | 3.20 | 9.72 | 32.79 | 0.11 | 0.11 |
| B | Tree5 | 1 | 1.48 | 16.51 | 34.64 | 2.86 | 2.79 | 1.46 | 0.98 | 0.95 | 6.16 | 17.00 | 0.70 | 1.94 | 4.33 | 48.21 | 3.59 | 10.90 | 32.72 | 0.13 | 0.12 |
| B | Tree5 | 2 | 1.39 | 15.65 | 33.19 | 3.21 | 3.13 | 1.64 | 1.10 | 1.06 | 6.07 | 15.31 | 0.79 | 2.18 | 0.30 | 55.27 | 4.51 | 7.55 | 31.97 | 0.14 | 0.26 |
| B | Tree6 | 1 | 1.32 | 14.51 | 32.02 | 3.81 | 2.67 | 1.33 | 0.95 | 1.09 | 1.67 | 25.46 | 0.66 | 1.43 | 3.86 | 50.21 | 3.20 | 9.72 | 32.79 | 0.11 | 0.11 |
| B | Tree6 | 2 | 1.25 | 13.30 | 30.97 | 4.28 | 2.99 | 1.50 | 1.07 | 1.22 | 1.87 | 24.60 | 0.74 | 1.60 | 4.31 | 51.20 | 2.98 | 7.93 | 33.16 | 0.25 | 0.16 |
| C | Tree1 | 1 | 2.40 | 15.24 | 37.31 | 0.29 | 1.80 | 0.73 | 0.62 | 0.41 | 2.00 | 25.59 | 0.60 | 1.32 | 4.83 | 47.30 | 3.34 | 8.90 | 35.16 | 0.28 | 0.18 |
| C | Tree1 | 2 | 1.69 | 14.52 | 36.69 | 0.33 | 2.02 | 0.82 | 0.69 | 0.46 | 2.25 | 24.45 | 0.68 | 1.48 | 0.50 | 51.65 | 4.35 | 7.82 | 35.08 | 0.42 | 0.17 |
| C | Tree2 | 1 | 2.31 | 21.30 | 24.37 | 4.25 | 2.14 | 1.00 | 0.77 | 0.77 | 1.67 | 25.60 | 0.79 | 1.34 | 0.57 | 49.44 | 4.90 | 8.80 | 35.63 | 0.48 | 0.19 |
| C | Tree2 | 2 | 2.01 | 21.05 | 24.08 | 4.77 | 2.41 | 1.12 | 0.86 | 0.86 | 1.88 | 26.76 | 0.89 | 1.51 | 1.61 | 65.42 | 3.95 | 7.56 | 20.91 | 0.35 | 0.19 |
| C | Tree3 | 1 | 1.35 | 34.75 | 12.55 | 0.50 | 2.94 | 0.99 | 0.69 | 1.33 | 1.90 | 26.39 | 0.73 | 0.68 | 1.81 | 64.26 | 4.43 | 8.48 | 20.41 | 0.40 | 0.21 |
| C | Tree3 | 2 | 1.88 | 33.50 | 11.10 | 0.56 | 3.30 | 1.11 | 0.77 | 1.49 | 2.14 | 25.86 | 0.82 | 0.76 | 1.68 | 60.83 | 2.76 | 7.63 | 26.66 | 0.18 | 0.25 |
| C | Tree4 | 1 | 2.18 | 11.05 | 50.47 | 1.59 | 1.76 | 0.18 | 0.71 | 0.61 | 1.24 | 15.75 | 0.42 | 0.99 | 1.89 | 59.53 | 3.11 | 8.58 | 26.40 | 0.21 | 0.28 |
| C | Tree4 | 2 | 2.06 | 10.42 | 49.70 | 1.79 | 1.98 | 0.20 | 0.80 | 0.69 | 1.40 | 14.69 | 0.47 | 1.11 | 1.14 | 55.22 | 3.84 | 6.12 | 33.29 | 0.11 | 0.27 |
| C | Tree5 | 1 | 1.86 | 16.83 | 40.37 | 1.66 | 2.00 | 0.46 | 1.01 | 0.36 | 1.66 | 18.65 | 0.62 | 1.40 | 1.29 | 53.60 | 4.33 | 6.90 | 33.45 | 0.13 | 0.31 |
| C | Tree5 | 2 | 1.59 | 15.91 | 39.56 | 1.87 | 2.25 | 0.51 | 1.14 | 0.41 | 1.86 | 17.39 | 0.70 | 1.57 | 3.21 | 62.17 | 3.41 | 6.52 | 24.38 | 0.15 | 0.16 |
| C | Tree6 | 1 | 1.30 | 32.78 | 17.89 | 1.13 | 1.68 | 0.22 | 0.62 | 0.94 | 1.55 | 23.94 | 0.82 | 1.20 | 3.62 | 60.42 | 3.84 | 7.35 | 24.42 | 0.17 | 0.18 |
| C | Tree6 | 2 | 1.28 | 31.82 | 16.61 | 1.27 | 1.88 | 0.25 | 0.70 | 1.05 | 1.75 | 22.90 | 0.92 | 1.35 | 0.30 | 61.27 | 4.93 | 8.47 | 24.52 | 0.22 | 0.28 |
| C | Tree7 | 1 | 1.95 | 12.85 | 45.68 | 3.17 | 1.63 | 0.16 | 0.78 | 0.71 | 1.50 | 17.18 | 0.63 | 1.11 | 0.34 | 59.58 | 5.53 | 9.51 | 24.47 | 0.25 | 0.32 |
| C | Tree7 | 2 | 1.88 | 11.54 | 44.73 | 3.56 | 1.83 | 0.18 | 0.87 | 0.80 | 1.68 | 16.30 | 0.71 | 1.25 | 3.62 | 60.42 | 3.84 | 7.35 | 24.42 | 0.17 | 0.18 |
| C | Tree8 | 1 | 1.18 | 26.55 | 29.81 | 0.30 | 2.31 | 0.99 | 1.11 | 0.54 | 1.20 | 17.48 | 0.65 | 1.22 | 3.03 | 47.65 | 3.39 | 8.34 | 37.11 | 0.10 | 0.38 |
| C | Tree8 | 2 | 1.82 | 25.78 | 28.49 | 0.33 | 2.59 | 1.11 | 1.2599 | 0.61 | 1.35 | 16.64 | 0.73 | 1.37 | 1.15 | 68.60 | 3.83 | 6.41 | 19.73 | 0.17 | 0.10 |
| C | Tree9 | 1 | 2.69 | 11.35 | 34.39 | 2.66 | 1.94 | 0.17 | 0.85 | 1.53 | 2.40 | 28.72 | 0.90 | 0.91 | 1.29 | 67.27 | 4.30 | 7.21 | 19.61 | 0.20 | 0.11 |
| C | Tree9 | 2 | 1.99 | 10.75 | 33.63 | 2.99 | 2.18 | 0.19 | 0.96 | 1.72 | 2.70 | 27.27 | 0.76 | 1.02 | 3.03 | 47.65 | 3.39 | 8.34 | 37.11 | 0.10 | 0.38 |
| C | Tree10 | 1 | 1.42 | 24.91 | 40.31 | 1.13 | 1.90 | 0.54 | 0.62 | 0.12 | 1.26 | 16.30 | 0.46 | 0.51 | 1.15 | 68.60 | 3.83 | 6.41 | 19.73 | 0.17 | 0.10 |
| C | Tree10 | 2 | 1.78 | 23.99 | 39.59 | 1.27 | 2.13 | 0.61 | 0.69 | 0.13 | 1.42 | 15.78 | 0.51 | 0.57 | 1.29 | 67.27 | 4.30 | 7.21 | 19.61 | 0.20 | 0.11 |

**Table 2** Data used for the Repeated Measures statistical analysis.

| Month | Tree | Rep | Plant | Oil yield | α-Pinene | p-Cymene | Limonene | α-Cedrene | β-Cedrene | Cedrol | Allo-cedrol | Monoterpenes | Sesquiterpenes |
| --- | --- | --- | --- | --- | --- | --- | --- | --- | --- | --- | --- | --- | --- |
| January | Tree1 | 1 | 1 | 0.9568 | 21.8263 | 1.48700 | 27.6700 | 2.78070 | 1.45850 | 30.4820 | 2.14900 | 76.3800 | 39.58 |
| January | Tree1 | 2 | 2 | 1.3636 | 20.7350 | 1.41265 | 26.2865 | 2.96417 | 1.73856 | 32.9579 | 2.04155 | 56.7761 | 42.27 |
| January | Tree2 | 1 | 3 | 0.7386 | 23.0348 | 0.90900 | 23.7150 | 1.83900 | 1.82100 | 32.5170 | 1.84200 | 58.5799 | 40.55 |
| January | Tree2 | 2 | 4 | 0.7580 | 21.7029 | 1.02717 | 24.7980 | 2.07807 | 1.60577 | 31.0744 | 2.08146 | 59.8689 | 39.70 |
| January | Tree3 | 1 | 5 | 0.7652 | 16.8080 | 1.43100 | 30.4720 | 1.94900 | 0.78700 | 34.3480 | 2.67500 | 57.3135 | 41.92 |
| January | Tree3 | 2 | 6 | 0.8085 | 15.4973 | 1.65996 | 32.3475 | 1.26084 | 0.91292 | 33.1814 | 2.10300 | 59.3203 | 39.96 |
| March | Tree1 | 1 | 1 | 0.6743 | 15.4500 | 1.88368 | 28.5394 | 3.15100 | 0.99100 | 36.7361 | 2.99827 | 52.9641 | 46.16 |
| March | Tree1 | 2 | 2 | 0.7631 | 16.0680 | 1.95903 | 29.6810 | 3.27704 | 1.03064 | 34.2021 | 3.11820 | 55.0826 | 44.01 |
| March | Tree2 | 1 | 3 | 0.6680 | 24.6510 | 0.94000 | 25.1660 | 2.57500 | 1.60200 | 31.4910 | 1.58000 | 58.7107 | 37.93 |
| March | Tree2 | 2 | 4 | 0.6803 | 26.9254 | 0.87420 | 27.4044 | 2.39475 | 1.48986 | 28.2866 | 1.46940 | 62.6009 | 36.67 |
| March | Tree3 | 1 | 5 | 1.2207 | 18.6310 | 1.38600 | 24.7250 | 3.05900 | 1.37300 | 33.8064 | 2.68800 | 56.1560 | 43.21 |
| March | Tree3 | 2 | 6 | 1.1076 | 17.6994 | 1.31670 | 25.4887 | 2.90605 | 1.30435 | 35.1708 | 2.55360 | 55.3482 | 44.10 |
| May | Tree1 | 1 | 1 | 0.4777 | 17.7130 | 1.82700 | 29.7391 | 1.74900 | 0.80200 | 28.3330 | 2.82700 | 61.5152 | 37.63 |
| May | Tree1 | 2 | 2 | 0.5617 | 18.0157 | 2.06451 | 28.1011 | 1.97637 | 0.90626 | 26.5016 | 3.19451 | 62.0080 | 37.01 |
| May | Tree2 | 1 | 3 | 0.5191 | 25.8990 | 1.01700 | 21.2710 | 2.46700 | 1.54100 | 29.5296 | 2.02400 | 59.2450 | 40.03 |
| May | Tree2 | 2 | 4 | 0.7784 | 23.5681 | 0.92547 | 23.3566 | 2.24497 | 1.40231 | 31.8719 | 1.84184 | 57.9129 | 41.43 |
| May | Tree3 | 1 | 5 | 0.6925 | 21.9560 | 1.63400 | 29.0560 | 2.75000 | 1.32600 | 26.7710 | 2.42700 | 63.8820 | 35.42 |
| May | Tree3 | 2 | 6 | 0.8708 | 23.4690 | 1.89544 | 26.7050 | 3.19000 | 1.53816 | 24.0544 | 2.81532 | 65.1031 | 34.09 |
| July | Tree1 | 1 | 1 | 0.2437 | 19.0560 | 1.57700 | 25.6660 | 1.77800 | 1.60900 | 31.7550 | 3.27500 | 58.0138 | 41.23 |
| July | Tree1 | 2 | 2 | 0.5760 | 17.8182 | 1.64008 | 27.6926 | 1.84912 | 1.67336 | 30.0252 | 3.40600 | 59.3344 | 39.87 |
| July | Tree2 | 1 | 3 | 0.5663 | 26.0990 | 0.99200 | 24.7930 | 2.79100 | 1.94790 | 26.4013 | 2.65500 | 62.1101 | 37.02 |
| July | Tree2 | 2 | 4 | 0.6579 | 27.2649 | 1.05152 | 25.2806 | 2.95846 | 2.06477 | 23.9009 | 2.81430 | 64.4367 | 35.15 |
| July | Tree3 | 1 | 5 | 0.6019 | 20.6300 | 1.42600 | 26.7385 | 3.31220 | 1.51900 | 30.6236 | 2.77300 | 58.9512 | 40.27 |
| July | Tree3 | 2 | 6 | 0.8673 | 19.5985 | 1.93547 | 25.4016 | 3.14659 | 1.44305 | 33.0924 | 2.63435 | 57.0276 | 42.26 |
| October | Tree1 | 1 | 1 | 0.3333 | 17.0410 | 1.60300 | 24.5240 | 1.78800 | 1.07400 | 35.7810 | 3.32200 | 55.0817 | 44.34 |
| October | Tree1 | 2 | 2 | 0.5812 | 19.2563 | 1.81139 | 22.1712 | 2.02044 | 1.21362 | 32.9325 | 3.75386 | 56.7014 | 42.61 |
| October | Tree2 | 1 | 3 | 0.4526 | 24.7400 | 0.91400 | 19.5220 | 2.68500 | 2.15800 | 33.7760 | 1.80000 | 54.7276 | 44.39 |
| October | Tree2 | 2 | 4 | 0.5678 | 23.0082 | 0.85002 | 21.1555 | 2.49705 | 2.00694 | 35.4117 | 1.67400 | 53.8967 | 45.28 |
| October | Tree3 | 1 | 5 | 0.5901 | 23.7600 | 1.39000 | 27.1324 | 2.98500 | 1.67000 | 28.2780 | 2.27000 | 61.4064 | 37.83 |
| October | Tree3 | 2 | 6 | 0.7900 | 21.1622 | 1.26490 | 28.6905 | 2.71635 | 1.51970 | 31.2733 | 2.06570 | 59.4204 | 39.96 |
| December | Tree1 | 1 | 1 | 0.6467 | 19.4770 | 1.64800 | 32.0760 | 1.89700 | 0.85000 | 28.6124 | 2.06300 | 62.7161 | 36.49 |
| December | Tree1 | 2 | 2 | 0.9278 | 16.8593 | 1.91168 | 34.2082 | 2.20052 | 0.98600 | 26.1904 | 2.39308 | 64.0167 | 35.33 |
| December | Tree2 | 1 | 3 | 0.9196 | 22.5690 | 0.95700 | 26.0616 | 2.64300 | 1.81800 | 30.5560 | 1.64000 | 57.6746 | 41.39 |
| December | Tree2 | 2 | 4 | 0.5117 | 23.9231 | 1.01442 | 25.2625 | 2.80158 | 1.92708 | 28.8389 | 1.73840 | 58.7723 | 40.33 |
| December | Tree3 | 1 | 5 | 0.9451 | 23.6380 | 1.75100 | 30.9597 | 3.32800 | 1.49300 | 19.9840 | 1.51500 | 69.9857 | 29.12 |
| December | Tree3 | 2 | 6 | 1.0775 | 24.5835 | 1.82104 | 32.0198 | 3.46112 | 1.55272 | 17.5783 | 1.57560 | 72.3871 | 27.08 |
