# Supplementary material for: Chemical profile of Juniperus excelsa M. Bieb. essential oil within and between populations and its weed seed suppression effect
Source: PLoS One. 2024 Feb 8;19(2):e0294126. doi: 10.1371/journal.pone.0294126 (PMC10852245; doi:10.1371/journal.pone.0294126)
Supplement: S1 Table — (PDF) [file pone.0294126.s004.pdf]

S1 Table Coordinates, meters above sea level (masl) in populations of *Juniperus excelsa* from Bulgaria.

Population	Coordinates	Masl	Tree number	Average % moisture
**The reserve "Tisata", part Maleshevska Mountain (MMT)**	41.74144° N, 023.15538° E	66	1	7.74
	41.74144° N, 023.15537° E	74	2	7.60
	41.74139° N, 023.15535° E	74	3	8.00
	41.74146° N, 023.15513° E	97	4	7.60
	41.74173° N, 023.15611° E	198	5	6.85
	41.74220° N, 023.15712° E	213	6	8.27
	41.74221° N, 023.15692° E	213	7	7.22
	41.73926° N, 023.155557°E	189	8	8.00
	41.74000° N, 023.15538° E	193	9	7.69
	41.74161° N, 023.15537° E	99	10	7.18
	41.74143° N, 023.15533° E	74	11	7.57
	41.74177° N, 023.15616° E	198	12	8.08
**The reserve "Tisata", part East Pirin Mountain (EPT)**	41.74004° N, 023.15536° E	366	1	8.15
	41.74004° N, 023.15537° E	366	2	10.01
	41.76820° N, 023.16172° E	362	3	8.07
	41.76830° N, 023.16165° E	362	4	8.66
	41.76841° N, 023.16170° E	365	5	10.66
	41.76855° N, 023.16168° E	359	6	13.33
**The reserve "Izgoryaloto Gyune", Krichim (IG)**	42°01′41.53″N, 24°28′12.83″E	331	1	20.67
	42°01′38.50″N, 24°28′10.03″E	346	2	43.16
	42°01′53.03″N, 24°28′06.13″E	354	3	28.22
	42°01′44.00″N, 24°28′08.12″E	360	4	31.53
	42°01′37.00″N, 24°28′09.23″E	352	5	23.23
	42°01′46.33″N, 24°28′06.08″E	369	6	21.63
	42°01′44.05″N, 24°28′38.08″E	411	7	33.39
	42°01′47.13″N, 24°28′09.83″E	329	8	9.01
	42°01′42.53″N, 24°28′10.83″E	359	9	14.64
	42°01′37.15″N, 24°28′08.07″E	366	10	14.55
