# Supplementary material for: Chemical profile of Juniperus excelsa M. Bieb. essential oil within and between populations and its weed seed suppression effect
Source: PLoS One. 2024 Feb 8;19(2):e0294126. doi: 10.1371/journal.pone.0294126 (PMC10852245; doi:10.1371/journal.pone.0294126)
Supplement: S2 Table — (PDF) [file pone.0294126.s005.pdf]

S2 Table. Average composition of *Juniperus excelsa* EOs from two populations from Bulgaria

**Compounds in %**	**RT**	**RI$_{calc}$**	**RI$_{lit}$**	**Locations**		
				**Reserve "Tisata"**		Reserve "Izgoryalot o gune", Krichim
				Malesjevska mountain	East Pirin	
				min±max	min±max	min±max
α-Thujene	9.48	928	924	0.08-0.28	0.07-0.27	0.06-0.21
α-Pinene	9.56	934	932	13.41-36.31	13.33-34.44	10.42-34.75
Camphene	9.93	943	946	0.06-0.45	0.11-0.29	0.07-0.37
Thuja-2,4(10)-diene	10.06	955	953	0.07-0.29	0.08-0.31	0.10-0.53
Sabinene	10.77	968	969	0.06-0.33	0.09-0.19	0.07-0.30
β-Pinene	10.83	971	974	0.18-0.52	0.15-0.83	0.15-0.56
Myrcene	11.28	986	988	0.11-0.97	0.22-0.96	0.33-1.25
δ-3-Carene	11.86	1010	1008	0.09-2.53	0.11-0.92	0.08-2.14
α-Terpinene	12.15	1015	1014	0.14-1.48	0.18-0.92	0.13-1.20
Limonene	12.77	1023	1024	14.60-40.74	12.79-34.64	11.10-50.47
γ-Terpinene	13.48	1053	1053	0.07-0.38	0.22-0.49	0.12-0.57
Terpinolene	14.33	1084	1086	0.09-0.47	0.12-0.44	0.12-0.51
p-Cymenene	14.42	1090	1089	0.08-0.29	0.10-0.28	0.09-0.47
β-Linalool	14.79	1098	1095	0.10-0.61	0.10-0.30	0.10-0.62
α-Fenchocamphorone	15.28	1105	1104	0.08-0.39	0.09-0.18	0.10-0.38
trans-Thujone	15.46	1113	1112	0.11-0.63	0.13-0.54	0.08-0.31
α-Campholenal	15.60	1124	1121	0.10-0.38	0.10-0.58	0.10-0.52
1-Terpineol	15.91	1131	1130	0.10-0.59	0.08-0.50	0.16-0.83
trans-Pinocarveol	16.14	1136	1135	0.11-0.97	0.10-0.35	0.10-0.51
cis-Verbenol	16.18	1138	1137	0.18-0.57	0.11-0.45	0.10-0.69
p-Mentha-1,5-dien-8-ol	16.97	1165	1166	0.08-0.93	0.10-0.28	0.10-0.31
1-Terpinen-4-ol	17.23	1177	1175	0.09-0.61	0.16-0.39	0.10-0.38
p-Cymen-8-ol	17.45	1180	1179	0.11-0.43	0.11-0.32	0.10-0.33
α-Terpineol	17.67	1184	1186	0.10-0.78	0.10-0.36	0.10-0.22
γ-Terpineol	18.00	1200	1199	0.11-0.39	0.10-0.36	0.12-0.55
trans-Carveol	18.37	1216	1216	0.10-0.35	0.16-0.30	0.12-0.39
cis-Carveol	18.74	1225	1226	0.07-0.47	0.10-0.30	0.13-0.66
Carvone	19.06	1241	1240	0.10-0.63	0.08-0.47	0.10-0.74
Piperitone	19.34	1250	1249	0.08-0.32	0.08-0.43	0.09-0.65
trans-Linalool oxide acetate	20.07	1290	1287	0.11-0.62	0.21-0.53	0.11-0.38
trans-2,4-Decadienol	21.09	1317	1319	0.13-1.93	1.53-4.28	0.29-4.77
α-Cubebene	21.92	1343	1345	0.11-0.26	0.11-1.74	0.10-1.13
α-Copaene	22.69	1374	1374	0.10-0.58	0.10-0.30	0.10-0.51
Sesquithujene	23.00	1403	1405	0.10-0.73	0.14-0.34	0.14-0.35
β-Caryophyllene	23.87	1418	1417	2.22-4.93	2.01-3.40	1.63-3.30

β-Cedrene	23.98	1421	1419	0.92-2.05	0.20-1.83	0.16-1.12
β-Copaene	24.12	1430	1430	0.10-1.12	0.13-1.06	0.16-1.11
Cedrane	24.24	1440	1441	0.11-2.85	0.30-1.27	0.15-0.84
epi-Cedrane	24.58	1444	1447	0.12-0.34	0.13-0.47	0.12-0.59
cis-Muurola-3,5-diene	24.64	1448	1448	0.11-0.36	0.18-0.65	0.11-0.54
α-Caryophyllene	24.75	1455	1452	0.17-0.47	0.20-0.80	0.10-0.54
Germacrene D	25.01	1480	1484	0.10-1.42	0.22-1.11	0.12-0.35
(E,E)-α-Farnesene	25.15	1506	1505	0.11-0.46	0.15-1.11	0.19-1.43
γ-Cadinene	25.23	1512	1513	0.10-1.60	0.25-0.97	0.14-0.63
δ-Cadinene	25.39	1521	1522	0.20-2.11	0.71-1.85	0.50-1.03
Dauca-4(11),8-diene	25.67	1529	1530	0.10-0.82	0.50-2.02	0.11-0.82
10-epi-Cubebol	25.75	1536	1533	0.15-1.68	0.35-1.20	0.13-1.82
cis-Muurol-5-en-4β-ol	26.38	1550	1550	0.14-1.04	0.44-2.75	0.17-1.82
Caryophyllene oxide	27.82	1583	1582	0.13-2.43	0.23-1.85	0.12-1.53
Allo-cedrol	28.20	1590	1589	1.57-2.47	1.28-6.47	1.20-2.70
Cedrol	28.70	1602	1600	2.94-35.15	15.31-29.83	14.69-28.72
1,10-di-epi-Cubenol	28.91	1617	1618	0.19-2.00	0.63-1.49	0.42-0.92
1-epi-Cubenol	29.02	1630	1627	0.11-1.79	0.14-0.39	0.13-0.83
tau.-Cadinol	29.11	1640	1640	0.14-0.58	0.22-0.59	0.11-0.80
tau.-Muurolol	29.16	1643	1644	0.21-0.88	0.11-0.45	0.13-0.90
Cubenol	29.24	1646	1645	0.31-2.87	0.40-2.87	0.51-1.48
