# Supplementary material for: Chemical profile of Juniperus excelsa M. Bieb. essential oil within and between populations and its weed seed suppression effect
Source: PLoS One. 2024 Feb 8;19(2):e0294126. doi: 10.1371/journal.pone.0294126 (PMC10852245; doi:10.1371/journal.pone.0294126)
Supplement: S3 Table — (PDF) [file pone.0294126.s006.pdf]

S3 Table Average composition of seasonal variability of *Juniperus excelsa* EOs

RT	RI_{lit}	RI_{calc}	Compound name	Month min-max between trees and repitition					
				January	March	May	July	October	December
9.39	922	924	α-Thujene	0.10-0.20	0.11-0.23	0.11-0.34	0.11-0.20	0.15-0.23	0.11-0.33
9.82	932	932	α-Pinene	15.50-23.03	15.45-26.93	17.71-25.90	17.82-27.26	17.04-24.74	16.86-24.58
10.24	943	945	α-Fenchene	0.12-0.30	0.18-0.78	0.31-0.48	0.10-0.40	0.17-0.50	0.12-0.68
10.29	947	946	Camphene	0.19-0.47	0.28-0.44	0.21-0.46	0.21-0.44	0.21-0.56	0.21-0.50
11.21	974	974	β-Pinene	0.17-3.63	0.14-0.37	0.23-0.49	0.23-0.34	0.14-0.26	0.19-0.32
11.63	987	988	β-Myrcene	0.16-0.38	0.12-0.27	0.15-0.36	0.15-0.22	0.14-0.21	0.18-0.42
12.24	1008	1008	δ-3-Carene	0.22-0.41	0.16-3.86	0.20-2.25	0.09-1.84	0.13-0.46	0.15-3.45
12.78	1019	1020	p-Cymene	0.91-1.66	0.87-1.96	0.93-2.06	0.99-1.94	0.85-1.60	0.96-1.91
13.01	1025	1024	Limonene	23.72-32.35	24.73-29.68	21.27-29.74	24.79-27.69	19.52-28.69	25.26-34.21
14.80	1083	1083	Fenchone	0.18-0.35	0.10-0.31	0.14-0.34	0.17-0.31	0.09-0.34	0.14-0.32
15.12	1097	1099	α-Pinene oxide	0.14-0.59	0.29-0.68	0.16-0.30	0.24-0.52	0.15-0.63	0.31-0.60
15.46	1111	1110	δ-Camphenol	0.28-0.53	0.11-0.33	0.18-0.58	0.25-0.46	0.30-0.48	0.23-0.35
15.99	1122	1121	α-Campholenal	0.29-0.49	0.20-0.35	0.33-0.79	0.20-0.57	0.33-0.86	0.23-0.52
16.17	1132	1132	(Z)-Limonene oxide	0.37-0.52	0.29-0.60	0.30-0.45	0.19-0.37	0.23-0.40	0.42-0.86
16.31	1137	1138	(E)-Limonene oxide	0.40-0.86	0.29-0.62	0.42-0.94	0.39-0.59	0.30-0.77	0.40-0.84
16.44	1140	1140	(E)-Verbenol	0.23-0.28	0.21-0.27	0.28-0.36	0.29-0.52	0.22-0.34	0.23-0.28
16.59	1152	1154	β-Pinene oxide	0.48-0.94	0.43-0.93	0.57-0.96	0.29-0.91	0.65-0.87	0.49-0.85
17.84	1182	1182	(Z)-Pinocarveol	0.23-0.48	0.24-0.68	0.24-0.76	0.26-0.67	0.36-0.55	0.23-0.60
18.08	1194	1195	Myrtenol	0.20-0.35	0.19-0.30	0.26-0.56	0.24-0.50	0.20-0.63	0.24-0.41
18.19	1199	1199	(E)-p-Menthan-2-one	0.26-0.42	0.12-0.29	0.27-0.56	0.21-0.42	0.25-0.48	0.22-0.40
18.40	1203	1204	Verbenone	0.33-0.40	0.18-0.34	0.27-0.70	0.23-0.63	0.28-0.56	0.18-0.45
18.77	1215	1216	(E)-Carveol	0.46-1.13	0.31-0.71	0.63-1.43	0.56-1.48	0.46-1.14	0.45-1.11

19.15	1226	1226	(Z)-Carveol	0.14-0.29	0.13-0.26	0.21-0.74	0.17-0.32	0.16-0.40	0.14-0.29
19.47	1239	1240	Carvone	0.47-1.12	0.36-0.72	0.64-1.82	0.52-1.40	0.40-1.56	0.48-1.10
21.89	1340	1339	(E)-Carvyl acetate	0.23-0.47	0.16-0.28	1.04-1.54	0.65-2.26	0.72-1.71	0.80-1.41
22.29	1365	1366	(Z)-Carvyl acetate	0.36-0.62	0.21-0.50	0.23-0.87	0.58-1.61	0.57-1.13	0.53-0.94
24.17	1410	1410	α-Cedrene	1.26-2.96	2.39-3.28	1.75-3.19	1.78-3.31	1.79-2.99	1.90-3.46
24.39	1419	1418	β-Cedrene	0.79-1.82	0.99-1.60	0.80-1.54	1.44-2.06	1.07-2.16	0.85-1.93
24.66	1440	1440	(Z)-β-Farnesene	0.39-0.89	0.29-0.47	0.27-1.04	0.28-1.00	0.63-1.49	0.78-1.59
24.87	1454	1455	(E)-β-Farnesene	0.22-0.70	0.53-1.44	0.60-1.45	0.52-1.11	0.45-1.02	0.77-1.63
28.61	1589	1590	Allo-cedrol	1.84-2.68	1.47-3.12	1.84-3.19	2.63-3.41	1.67-3.75	1.52-2.39
29.01	1600	1600	Cedrol	30.48-34.35	28.29-36.74	24.05-31.87	23.90-33.09	31.27-35.78	17.58-30.56
29.30	1627	1627	1-epi-Cubenol	0.44-1.05	0.25-0.75	0.46-1.50	0.22-1.24	0.20-1.03	0.19-0.89
29.68	1644	1644	tau.-Cadinol	0.26-0.45	0.33-0.56	0.25-0.69	0.26-0.33	0.18-0.30	0.26-0.39
29.94	1652	1652	tau.-Muurolol	0.21-0.61	0.27-0.40	0.34-0.73	0.21-0.58	0.14-0.49	0.18-0.96
