# Supplementary material for: Chemical profile of Juniperus excelsa M. Bieb. essential oil within and between populations and its weed seed suppression effect
Source: PLoS One. 2024 Feb 8;19(2):e0294126. doi: 10.1371/journal.pone.0294126 (PMC10852245; doi:10.1371/journal.pone.0294126)
Supplement: S4 Table — (PDF) [file pone.0294126.s007.pdf]

S4 Table. Compounds of concrete and resinoid of *Juniperus excelsa*

**Compounds %**	**RI$_{calc}$**	**RI$_{lit}$**	**RT**	***Juniperus excelsa***	
				**concrete**	**resinoid**
(E)-Verbenol	1140	1141	16.57	0.13	nd
(2E-4E)-Decadienol	1319	1321	21.39	0.28	0.98
β-Cedrene	1419	1420	24.16	0.58	1.24
Cubebol	1514	1515	26.64	0.64	0.34
(E)-Calamenene	1521	1522	26.77	0.21	0.41
Dodecanoic acid (Lauric acid)	1565	1566	27.76	0.20	0.88
Allo-cedrol	1590	1589	28.60	1.04	1.82
Cedrol	1600	1600	28.99	15.39	28.15
epi-Cedrol	1618	1619	29.29	0.27	0.62
5-Cedranone	1628	1630	30.08	1.00	1.71
Junicedranone	1664	1665	30.68	0.24	1.27
4-hydroxy-Coumarin	1794	1795	31.95	nd	5.83
7-hydroxy-Coumarin	1836	1837	34.35	nd	3.10
7-hydroxy-4-methyl-Coumarin	1981	1980	39.52	17.63	26.94
7-hydroxy-4.8-dimethyl-Coumarine	2013	2013	40.13	0.66	1.41
n-Heneicosane	2100	2100	40.75	nd	1.96
Abienol	2149	2150	42.34	0.27	1.26
dehydro-Abietal	2274	2275	43.26	0.20	0.75
4-epi-Abietal	2298	2290	43.36	0.24	2.14
4-epi-Abietol	2343	2344	44.01	0.47	1.63
dehydro-Abietol	2368	2367	44.13	0.53	1.89
Abietol	2401	2400	44.76	0.32	3.84
Hexacosane	2600	2600	47.02	nd	2.61
1-Octacosanol	3118	3120	54.21	36.85	6.32
Dotriacontane	3200	3200	54.74	nd	1.07
Tritriacontane	3300	3300	56.80	16.08	nd
Triacontanoic acid (Melissic acid)	3344	3342	57.04	4.79	nd
Tetratriacontane	3400	3400	58.24	1.41	1.01
